# Paternal overweight is associated with increased breast cancer risk in daughters in a mouse model

**DOI:** 10.1038/srep28602

**Published:** 2016-06-24

**Authors:** Camile Castilho Fontelles, Elissa Carney, Johan Clarke, Nguyen M. Nguyen, Chao Yin, Lu Jin, M. Idalia Cruz, Thomas Prates Ong, Leena Hilakivi-Clarke, Sonia de Assis

**Affiliations:** 1 Department of Oncology, Lombardi Comprehensive Cancer Center, Georgetown University, Washington, DC, USA; 2 Departament of Food and Experimental Nutrition, Faculty of Pharmaceutical Sciences, University of São Paulo and Food Research Center, São Paulo, SP, Brazil

## Abstract

While many studies have shown that maternal weight and nutrition in pregnancy affects offspring’s breast cancer risk, no studies have investigated the impact of paternal body weight on daughters’ risk of this disease. Here, we show that diet-induced paternal overweight around the time of conception can epigenetically reprogram father’s germ-line and modulate their daughters’ birth weight and likelihood of developing breast cancer, using a mouse model. Increased body weight was associated with changes in the miRNA expression profile in paternal sperm. Daughters of overweight fathers had higher rates of carcinogen-induced mammary tumors which were associated with delayed mammary gland development and alterations in mammary miRNA expression. The hypoxia signaling pathway, targeted by miRNAs down-regulated in daughters of overweight fathers, was activated in their mammary tissues and tumors. This study provides evidence that paternal peri-conceptional body weight may affect daughters’ mammary development and breast cancer risk and warrants further studies in other animal models and humans.

While the genome is relatively stable throughout an organism’s life span, the epigenome is malleable to ensure short-term adaptation to the environment^1^. Epigenetic plasticity is highest in early development and the risk of several chronic diseases in adulthood is thought to result from environmental exposures acting in early life^2^,^3^.

Most of the evidence for developmental environmental exposures on offspring’s disease risk comes from maternal exposures in pregnancy. However, the role of environmentally-acquired epigenetic alterations in the male germ-line and disease susceptibility in their progeny has been actively investigated in the past few years in both humans^4^,^5^ and animal models^6^,^7^,^8^,^9^,^10^,^11^,^12^,^13^. Male primordial germ cells are first produced during embryonic development and their maturation process starts before puberty and spans the entire reproductive life^14^,^15^,^16^. It has been proposed that environmentally-induced epigenetic modifications of the male germline coincides with those three life stages^12^.

In mammals, epigenetic information has been shown to be transmitted from one generation to another through the germ-line and carry environmental epigenetic memory from the previous generation to the next^10^,^17^,^18^. Epigenetic information in cells is carried via three different, but closely interacting, mechanisms: DNA methylation, histone modifications and non-coding RNAs. This machinery is responsible for regulating gene expression and cellular differentiation during embryonic development and throughout life^14^,^19^,^20^.

It is well-established that maternal exposures (e.g. nutrition, endocrine disruptors) in pregnancy can result in changes in mammary tissue development and breast cancer risk in the offspring in both humans and rodent studies^21^,^22^,^23^. Further, in animal models, *in utero* nutritional and estrogenic exposures increases mammary cancer risk that can be inherited by subsequent generations likely through epigenetic mechanisms^18^. Birth weight, believed to be a marker of in *utero* nutrition, is positively and consistently associated with breast cancer risk in population studies^24^ as well as animal studies^25^. However, birth weight in the offspring can also be determined by the father’s peri-conceptional body weight^26^.

Here, we sought to determine the contribution of paternal consumption of an obesity-inducing diet and ensuing overweight around the time of conception on their daughters’ mammary development and breast cancer risk, using a carcinogen-induced mouse model of this disease. We found that peri-conceptional overweight in males is associated with high birth weight, delayed mammary gland maturation and increased mammary cancer risk in their female offspring. We also observed that increased body weight led to miRNA profile alterations in paternal sperm and in their daughters’ mammary tissue. Some of the altered miRNAs regulate genes commonly deregulated in cancer, particularly, those involved in the hypoxia signaling pathway, which was altered in both mammary tissue and mammary tumors of overweight fathers’ offspring.

## Results

### Effect of dietary consumption on paternal body weight and sperm miRNA expression profile

**Figure 1:**
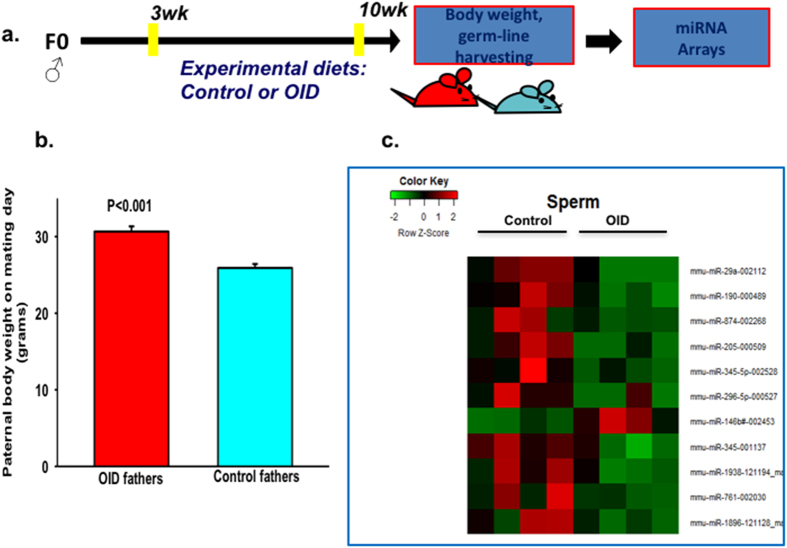
Effects of OID consumption on paternal body weight and germline microRNA expression profile. (**a**) Experimental design, (**b**) OID and control male mice body weight at 10 weeks of age, (**c**) Heatmap of miRNA expression profile in spermatozoa of OID fathers compared to controls (n = 4/group). Body weight data (n = 11/group) are mean ± s.e.m. and significant differences versus the control group assessed by t-test. *P* < 0.05 is considered significant; exact *P* values are shown in each plot. MicroRNA expression levels were assessed by TaqMan^®^ MicroRNA Assay.

Male mice (*c57bl/6*) consumed either a control or an obesity-inducing diet (OID) from 3 to 10 weeks of age (Experimental design, Fig. 1a and Figure S1). OID consumption by male mice led to an increase in body weight compared with control males (*p* < 0.001, Fig. 1b, Table S1).

Recent studies suggest that the male germline can be epigenetically reprogrammed due to life-style and environmental exposures in humans^27^ and animals^9^,^10^. To explore the long-term effects of paternal overweight on miRNA expression levels, we performed miRNA array experiments using RNA extracted from mature sperm. We found that, compared with controls, OID males had 11 differentially expressed miRNAs, of which ten were down-regulated and one was up-regulated (Fig. 1c, Table S2).

### Effects of paternal overweight on offspring’s birth outcomes

**Figure 2:**
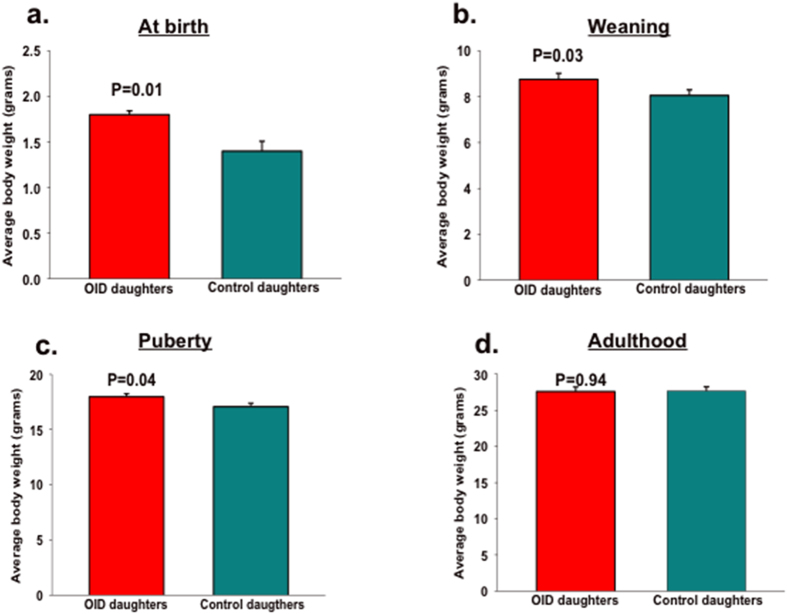
Effect of paternal overweight on female offspring’s body weight at different stages of life. Body weight (g) of OID offspring (**a**) at birth, (**b**) at weaning (pre-puberty), (**c**) in post-puberty and (**d**) in adulthood. All data (n = 21–33/group) are mean ± s.e.m. Significant differences versus the control group assessed by t-test. *P* < 0.05 is considered significant; exact *P* values are shown in each plot.

Control and OID male mice were then mated with female mice reared on the control diet. Paternal overweight at the time of conception increased birth weight (Fig. 2a) of female offspring (*p* = 0.013). Paternal overweight also increased birth weight of male offspring, but it did not reach statistical significance (*p* = 0.07). The increase in female offspring’s body weight persisted through puberty onset (*p* = 0.036, Fig. 2b,c), but differences in body weight between the two groups disappeared as animals grew older, i.e. at the end of the tumor monitoring period (Fig. 2d, *p* = 0.94).

### Effects of paternal overweight on female offspring’ mammary gland development

**Figure 3:**
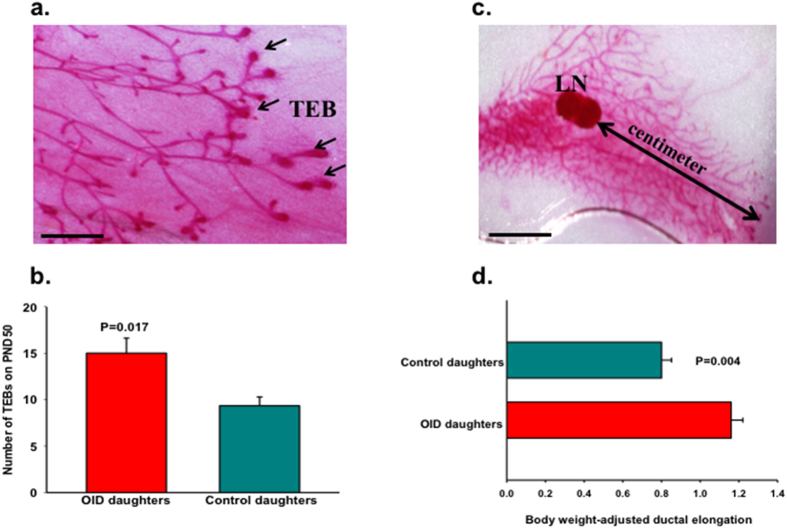
Effect of paternal overweight on female offspring’s mammary gland differentiation. (**a**) Histological depiction of the 4^th^ inguinal mouse mammary gland on PND 50 showing terminal end buds (TEB, indicated by arrows) and (**b**) number of TEBs (n = 5–6/group) on PND50 in control and OID female offspring. (**c**) Histological depiction of the 4^th^ inguinal mouse mammary gland on PND 50 showing ductal elongation (indicated by arrow, cm) and **(d**) body weight-adjusted ductal elongation (cm, n = 5–6/group)) on PND50 in control and OID female offspring. All values are expressed as the mean ± s.e.m. Significant differences versus the control group assessed by t-test. *P* < 0.05 is considered significant; exact *P* values are shown in each plot. LN, lymph node; scale bars: 0.5 mm (**a**) and 3 mm (**c**). Mammary tissues analyzed represent litters from 3–4 different fathers/group.

Birth weight has been shown before to modulate mammary gland development^25^. In order to determine the effects of high birth weight and ancestral overweight on mammary gland development, we analyzed normal mammary tissue of female offspring after puberty onset, on post-natal day (PND) 50. This is also when the mammary tissue is most susceptible to carcinogen induced initiation of breast cancer in rodents. The number of terminal end buds (TEBs), undifferentiated mammary structures which are the targets for malignant transformation^28^ was significantly increased (*p* = 0.017, Fig. 3a,b, Table S3) in OID daughters. Mammary ductal elongation, adjusted by body weight, was also higher in the OID female offspring ((*p* = 0.004, Fig. 3c,d) compared to controls.

### Effects of paternal overweight on mammary tumorigenesis in female offspring

**Figure 4:**
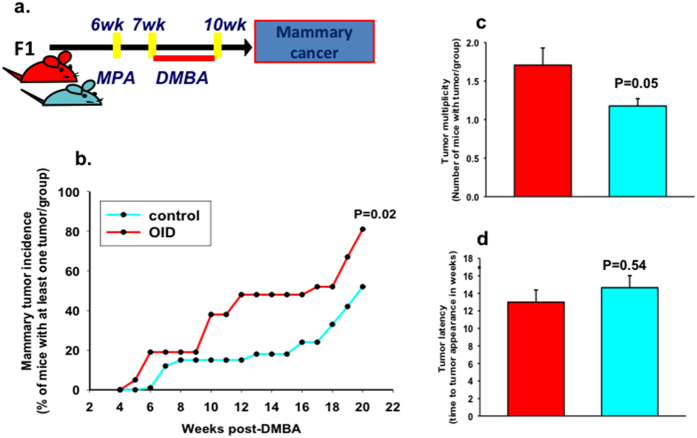
Effect of paternal overweight on female offspring’s mammary tumorigenesis. (**a**) Experimental design, (**b**) Mammary tumor incidence (%) in control and OID female offspring (n = 21–33/group,). (**c**) Mammary tumor multiplicity (mean ± s.e.m.) in control and OID female offspring. (**d**) Mammary tumor latency (mean ± s.e.m.) in control and OID female offspring. Significant differences versus the control group were determined as follows: log-rank test (tumor incidence) and t-test (tumor multiplicity and latency). *P* < 0.05 is considered significant; exact *P* values are shown in each plot.

Because of the observed alterations in mammary gland development, we next studied the mammary cancer risk of OID female offspring, using a well-established^29^,^30^,^31^ carcinogen-induced mouse model of breast cancer (Fig. 4a). The incidence of palpable mammary tumors in the OID female mice was significantly higher than in controls (*p* = 0.02, Fig. 4b). Tumor multiplicity was also higher in OID offspring (*p* = 0.052, Fig. 4c), but did not reach statistical significance. There were no differences in tumor latency between the groups (*p* = 0.54, Fig. 4d). All tumors included in our analyses were determined to be malignant mammary carcinomas by a pathologist.

### Effect of paternal overweight on offspring’s mammary tissue microRNA expression profile

**Figure 5:**
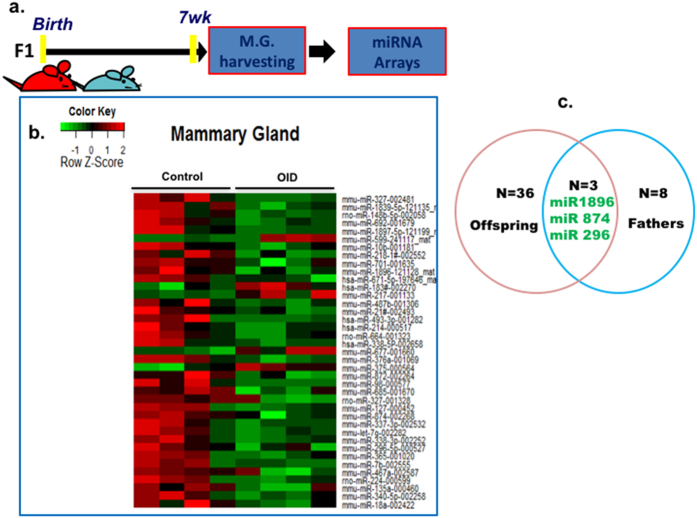
Effect of paternal overweight on female offspring’s mammary tissue microRNA expression profile. (**a**) Experimental design (**b**) Heatmap of miRNA expression profile in normal mammary tissue of OID offspring compared to controls (n = 4/group, representing litters from 3 different fathers/group). MicroRNA expression levels were assessed by TaqMan^®^ MicroRNA Assay (**c**) Venn diagram of paternal sperm and offspring’s mammary tissue showing the three miRNAs significantly down-regulated in both groups.

Deregulation of miRNA expression is important in the pathogenesis of breast cancer^32^,^33^. Thus, we examined whether alterations in mammary gland development and cancer risk were associated with changes in mammary miRNA expression in OID female offspring using miRNA arrays (Fig. 5a). Compared with controls, OID female offspring had a total of 39 differentially expressed miRNAs in their mammary glands, five of which were up-regulated and thirty-four down-regulated (Fig. 5b, Table S4).

A comparison between the miRNAs expression profile of OID daughters and their fathers revealed that three miRNAs were similarly altered in both generations (Fig. 5c): mmu-miR-1896, mmu-miR-874 and mmu-miR-296-5p were down-regulated in paternal sperm and their female offspring’s mammary tissue.

### Enrichment analysis

We focused our down-stream analyses on mmu-miR-1896, mmu-miR-874 and mmu-miR-296-5p. It is estimated that a single miRNA can target hundreds of gene transcripts^34^. We performed a gene set enrichment analysis using the predicted target genes for murine miRNAs 1896, 874 and 296-5p and the top ten canonical pathways generated are shown in Table 1. A complete list of pathways is shown in Table S5. In addition, a list of canonical pathways generated using predicted targets for each individual miRNA is provided in Table S6 (mmu-miR-1896), Table S7 (mmu-miR-874), and S8 (mmu-miR-296-5p).

### Validation of target genes expression in mammary tissue and mammary tumors

We then set out to analyze and validate the expression of members of some of the pathways uncovered in the enrichment analysis in mammary tissue and tumors of OID daughters using western-blot or immunohistochemistry. We focused on the following pathways: ER α, ERK/MAPK, Hypoxia signaling, and Epithelial to Mesenchymal Transition (EMT). We observed no change in the expression of ER- α, but detected higher levels of MAPK activity in OID mammary tissue than in the controls. Neither of those pathways was altered in OID mammary tumors (Figure S2).

**Figure 6:**
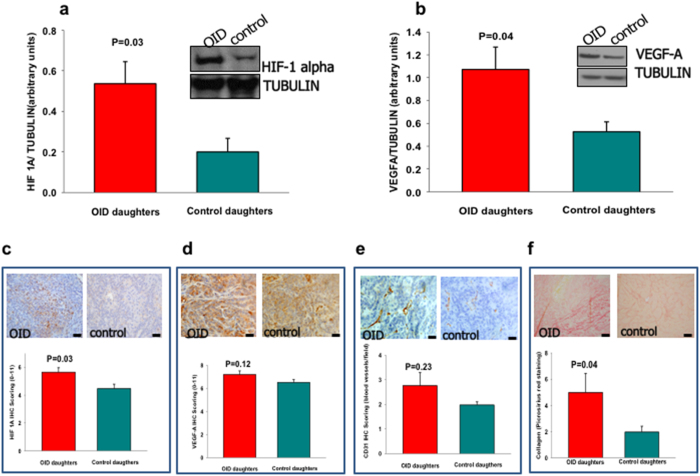
Validation of signaling pathways alterations associated with miR-874, 296-5p, and 1896 down-regulation in normal mammary tissue and mammary tumors of OID dautgters. (**a,b**) Representative western-blot bands and quantification of (**a**) HIF-1alpha and (**b**) VEGF-A expression on PND50 mammary tissue of control and OID offspring. (**c–f**), Representative mammary tumor sections (40X magnification) and staining scores for (**c**) HIF-1 alpha (dark nuclei), (**d**) VEGF-A, (**e**) CD31 staining (blood vessels) and (**f**) collagen fibers (Sirius Red). All data are mean ± s.e.m (n = 4–6/group). Significant differences versus the control group assessed by t-test. *P* < 0.05 is considered significant; exact *P* values are shown in each plot. Scale bars: 20 μm.

A member of the hypoxia signaling and EMT pathways, HIF-1α, was up-regulated in mammary tissue of the OID offspring compared with controls (*p* = 0.03, Fig. 6a). The levels of HIF-1α’s down-stream target, VEGF-A, were also higher in OID mammary tissues (*p* = 0.04, Fig. 6b). Further, we observed higher levels of HIF-1α expression in OID mammary tumors compared with control tumors (*p* = 0.03, Fig. 6c). However, while levels of VEGF-A were higher in OID tumors, this difference did not reach statistical significance (*p* = 0.12, Fig. 6d).

### Angiogenesis and epithelial to mesenchymal transition in OID mammary tumors

Because of the robust increase in HIF-1α and, to a less extent, VEGF-A expression in OID daughters’ normal mammary tissue and tumors, we investigated the levels of angiogenesis in mammary tumors using CD31 as a marker of endothelial cells. The levels of CD31 were slightly higher, but not significantly, in OID mammary tumors (*p* = 0.23, Fig. 6e).

HIF-1α has also been shown to promote stimulation of EMT via fibrogenesis: Under hypoxic conditions, HIF-1α up-regulates the expression of hydroxylases which regulate collagen hydroxylation, a step necessary for proper collagen folding and secretion^35^. Using Sirius Red Staining as a marker for collagen fibers, we were able to determine that tumors of OID daughters had higher levels of intra-tumoral collagen fibers than their control counterparts (*p* = 0.039, Fig. 6f).

## Discussion

It is well-established that maternal nutrition and weight gain in pregnancy modulate offspring’s breast cancer risk later in life^23^,^24^,^25^. In this study, we found that paternal overweight around the time of conception was associated with epigenetic changes in the father’s germline and with higher birth weight, altered mammary gland development and increased mammary cancer risk in their female offspring. In addition, we showed that the increased mammary cancer risk in OID female offspring was associated with changes in miRNA expression and target genes. To our knowledge, ours is one of the first studies to show, in an animal model, that male ancestral body weight can modulate the risk of breast cancer in their progeny.

The extent by which body weight-induced epigenetic changes in male germline influence mammary gland development in their progeny is currently unknown. Our data, however, suggest that ancestral paternal overweight is associated with changes in the offspring’s mammary tissue with consequences for its maturation and susceptibility to malignancy. Whether only local mammary tissue changes or other systemic alterations in OID daughters are important in determining that phenotype needs to be further explored. Previous literature reports^6^,^36^, however, show that paternal overweight can lead to metabolic changes in the offspring suggesting that systemic changes may also be important.

Locally, non-coding RNAs have been shown to play an important role in mammary gland development^37^ and tumorigenesis^38^. In our studies, we found that, addition to miRNA changes, mammary tissue of OID offspring had higher number of terminal end buds, undifferentiated units which are the targets for malignant transformation in rodents^28^. Further, OID offspring’s mammary tissue had extended ductal elongation, suggesting that the epithelial tissue has a higher proliferation potential. This is supported by higher activity of MAPK, a regulator of cell proliferation in the normal mammary tissue. However, whether alterations in mammary miRNA profile are functionally responsible for those morphological changes needs to be further investigated.

While no studies in humans have directly investigated the link between paternal body weight and breast cancer in their descendants, a few studies have examined the association between other paternal environmental exposures and cancer risk in the offspring. For instance, paternal age is linked to risk of breast cancer^39^ as well as hematological malignancies^40^ in their children. Paternal ethnicity (which can be an indicator of both genetic background as well as environmental exposures) has also been linked to risk of breast cancer in daughters^41^. Paternal cigarette smoking is associated with childhood cancers^42^.

Although the evidence for the contribution of paternal life-style to offspring’s cancer risk is limited, several studies suggest that paternal experiences before and around the time of conception affects other health outcomes in their progeny in both humans and animal models. In rodents, consumption of a high-fat diet or a low-protein diet before conception leads to metabolic abnormalities in offspring^6^. Two recent studies in mice show that the male germline can transmit the effects of early trauma or stress to the next generation^9^,^10^. In humans, male peri-pubertal nutrition can alter their progeny’s risk of diabetes, cardiac disease and overall mortality^5^ and grandsons’ longevity^4^.

Three major stages of development have been proposed in which the male germline can undergo epigenetic alterations: gonadal sex determination, the peri-pubertal and pubertal stages, and the peri-conceptional periods^12^. Our findings are in agreement with other reports showing that environmental insults can alter the male germline via epigenetic mechanisms (DNA methylation, non-coding RNAs, histone modifications^9^,^10^,^27^,^43^). For instance, paternal exposure to cyclophosphamide leads to chromatin remodeling in rat spermatozoa^44^. In humans, high BMI leads to epigenetic alterations in the male germline^27^ and is associated with differentially methylated regions in imprinted genes (*IGF-2*, *MEST*, *PEG3* and *NNAT*) in newborns^45^,^46^. Also in humans, paternal smoking alters miRNA expression in spermatozoa^43^. Further, the effects of trauma and stress are mediated through sperm miRNAs in mice^9^,^27^.

Small non-coding RNAs are present in sperm^47^,^48^ and can be delivered to the oocyte and regulate embryogenesis^49^. Studies in lower organisms^50^,^51^ and mice implicate them in non-Medelian inheritance of environmentally induced traits acquired in life^9^,^27^,^52^,^53^,^54^,^55^. Using zygote microinjections, Rodgers and colleagues^10^ showed that miRNAs altered by paternal stress recapitulate the offspring stress phenotype in a mouse model.

Our study, however, does not directly investigate the mechanisms by which ancestral body weight information is transmitted to the offspring and modulate cancer risk. While we observed that miRNAs 1896, 874 and 296-5p were regulated in the same direction in OID males’ sperm and their progeny’s mammary tissue, we have no evidence to support that those patterns of expression were epigenetically or otherwise inherited by OID daughters. At this stage, we can only conclude that there is an association and any causal relationship between those three miRNAs and epigenetic transmission of cancer risk is beyond the scope of this study and will require additional functional experiments. It is also possible that other small RNA species such as tRNA fragments^54^,^55^ or other epigenetic mechanisms such as DNA methylation play a role and need to be considered in future studies.

Deregulation of miRNA expression has been shown to be important in the pathogenesis of breast cancer^32^. miRNAs can act as either tumor suppressors or as oncogenes (oncomiRs) depending on which target genes they regulate^33^. HIF-1α, one of the predicted targets revealed in our enrichment analysis, is increased in OID daughters’ mammary tissue and tumors compared with controls. Increased HIF-1α not only promotes angiogenesis, it has been shown to promote EMT through regulation of collagen hydroxylation, a step necessary for proper collagen folding and secretion^56^. Increased EMT activity consequently promotes tumor progression by facilitating cancer cell migration and invasion^57^. Given its major role in tumor cell adaptation, HIF-1α has been shown to be a predictor of poor prognosis in breast and other cancers^57^,^58^. Thus, our findings suggest that paternal overweight is not only associated to increased breast cancer risk in their daughters, it may also lead to increased tumor aggressiveness and warrants further investigation.

In summary, our study shows that paternal peri-conceptional body weight can influence daughters’ birth weight, mammary gland development and breast cancer risk. Our study adds to an increasing number of studies suggesting that paternal environmental exposures are likely as important as maternal exposures in influencing health outcomes in the offspring. Whether paternal overweight itself (and underlying metabolic abnormalities) or specific components of an obesity-inducing diet are responsible for the phenotypes observed in OID daughters needs to be investigated using other models of obesity and isocaloric diets. While many questions still remain, if confirmed in humans, our findings could have important implications. First, our results support the notion that the familial or heritable aspect of breast cancer can be due not only to genetic factors but could also result from ancestral exposure and possibly inherited through modifiers of gene expression such as epigenetic mechanisms^15^. Whether the miRNAs we identified in our study play a role in increasing breast cancer risk still remains to be determined. Second, our findings indicate that life style-induced modifications in the adult male germline may play an important role and corroborate that the effects of overweight/obesity on cancer risk go beyond the directly exposed individual^6^,^24^. Finally, our findings could help explain health disparities in breast cancer outcomes. One example is that, while breast cancer prevalence is similar in Caucasians and African Americans, breast cancers diagnosed in African Americans tend to be more aggressive and have poorer prognosis^59^. Obesity is more prevalent in minority populations^60^ and ancestral weight and dietary patterns may play an important role in determining the breast cancer risk outcomes in women in those populations and needs to be further investigated.

## Experimental Procedures

### Breeding and dietary exposures

The *c57bl/6* strain of mice was used in all experiments. Male mice were fed AIN93G-based diets containing either 17% (control, n = 11) or *58*% energy from fat (Obesity-Inducing-Diet, OID, n = 11) (Table S9) starting after weaning (3 weeks of age). Males’ body weight was recorded weekly. At 10 weeks of age, OID-fed and control-fed male mice were mated to female mice reared on the control diet to generate the female offspring. Males were kept in female cages for 3 days. Female mice were kept on the control diet during the breeding period, for the extent of pregnancy (21 days) and after giving birth. The birth weight of pups and number of pups per litter was determined. To avoid litter-effect, pups were cross-fostered one day after dams give birth. Pups from 2–3 dams were pooled and housed in a litter of 8–10 pups per nursing dam. All pups were weaned on postnatal day 21 and fed the control diet throughout the experiment. Pups body weight were recorded weekly. The female offspring of control or OID fathers were used to study birth weight, mammary gland morphology, molecular analyses and mammary tumorigenesis as described in the following sections. All animal procedures were approved by the Georgetown University Animal Care and Use Committee, and the experiments were performed following the National Institutes of Health guidelines for the proper and humane use of animals in biomedical research.

### Mature spermatozoa collection and purification

Control and OID fathers were euthanized once mating was completed and caudal epididymis (for sperm collection) dissected. The cauda and vas deferens from male mice were collected, punctured, and transferred to tissue culture dish containing M2 media (M2 Medium-with HEPES, without penicillin and streptomycin, liquid, sterile-filtered, suitable for mouse embryo, SIGMA, product #M7167) where it was incubated for 1 hour at 37 °C. Sperm samples were isolated and purified from somatic cells following the protocol described by Goodrich *et al*.^61^. Briefly, the samples were washed with PBS, and then incubated with SCLB (somatic cell lysis buffer, 0.1% SDS, 0.5% TX-100 in Diethylpyrocarbonate water) for 1 hour. SCLB was rinsed off with 2 washes of PBS and the purified spermatozoa sample pelleted and used for miRNA extraction.

### Mammary gland harvesting

Inguinal mammary glands (4^th^ pair) of female offspring of control and obese fathers (n = 5–6 per group/age) were collected on postnatal days (PND) 50 and used for mammary gland development analysis and miRNA and protein extraction. Litters from 3–4 different fathers were represented in each of those analyses.

### microRNA arrays analysis

Total RNA from paternal sperm and their female offspring mammary tissue was extracted using miRNeasy kit (Qiagen, Valencia, CA) according to manufacturer’s instructions. RNA samples was quantified and stored at −80 °C until use.

MicroRNA arrays were performed at the Genomics and Epigenomics Shared Resources (GESR) at Georgetown University using Applied Biosystems TaqMan Rodent MicroRNA Arrays to generate the miRNA expression profile for each from experimental group. The TaqMan^®^ Array Rodent MicroRNA A+B Cards Set v3.0 is a two card set containing a total of 384 TaqMan^®^ MicroRNA Assays per card (Contains homologous mouse, rat and human detectors. Because they are highly conserved between species, all human assays can detected rodent miRNAs). The set enables accurate quantitation of 641 unique microRNAs for mouse. There are three TaqMan^®^ MicroRNA Assay endogenous controls for each species on each array to aid in data normalization, geNorm algorithm^62^ was applied to those endogenous control to determine the optimal number of stable controls. Geometric mean of those selected controls was used for array normalization. Statistical analysis was conducted by R package limma^63^. miRNA that has fdr < 0.1 were considered as significantly regulated miRNA which were then selected for further analysis.

Target prediction for microRNAs of interest was conducted using TargetScan (Release 6.2). The predicted targeted mRNA list was then uploaded to Ingenuity pathway analysis (IPA) for gene set enrichment analysis. We selected top 10 canonical pathways for further analysis.

### Mammary gland development

Inguinal mammary glands (4^th^ pair) were stretched onto a slide, placed in a fixative solution and stained with a carmine aluminum solution (Sigma Chemical Co.) as previously described^64^. Whole mounts were examined under the microscope and ductal elongation and number of TEBs (undifferentiated structure considered to be the targets of malignant transformation), as previously described^28^,^64^. All the analyses were blindly performed by two independent observers. Results were statistically tested using a t test (or corresponding non-parametric test).

### Mammary tumorigenesis

Mammary tumors were induced in female mice offspring (n = 21–33/group) by administration of MPA at 6 weeks of age, followed by four weekly doses of 1 mg of 9,12-dimethylbenz[a]anthracene (DMBA) (Sigma, St. Louis, MO) dissolved in peanut oil by oral gavage. This established model of breast cancer has been used by us and others^29^,^30^,^31^. Mice were examined for mammary tumors by palpation once per week, starting on week two after the last dose of DMBA and continue for a total of 20 weeks. Tumor growth was measured using a caliper and the width and height of each tumor were recorded. The end-points for data analysis were (i) latency to tumor appearance, (ii) the number of animals with tumors (tumor incidence), and (iii) the number of tumors per animal (tumor multiplicity). During follow-up, those animals in which tumor burden approximated 10% of total body weight were sacrificed, as required by our institution. Histopathology of tumors was evaluated commercially by Animal Reference Pathology (Salt Lake City, Utah). Differences in tumor latency and multiplicity were tested t-test (or corresponding non-parametric test). Kaplan-Meier survival curves were used to compare differences in tumor incidence, followed by the log-rank test.

### Analysis of protein levels in OID and control offspring’s mammary tissues and mammary tumors

#### Western blots

Protein levels of 50 ng/μL were assessed by western blot in mammary tissues obtained from OID or control female offspring (n = 4–6 per group). Total protein was extracted from mammary tissues using RIPA buffer with protease inhibitor (Roche, Switzerland), glycerophosphate (10 mM), sodium orthovanadate (1 mM), pyrophosphate (5 mM) and PMSF (1 mM). Protein extracts were resolved on a 4–12% denaturing polyacrylamide gel (SDS-PAGE). Proteins were transferred using the iBlot^®^ 7-Minute Blotting System (Invitrogen, USA) and blocked with 5% non-fat dry milk for 1 h at room temperature. Membranes were incubated with the specific primary antibodies (for antibody specifications and dilutions see Table S10) at 4 °C overnight. After several washes, the membranes were incubated with horseradish peroxidase (HRP)-conjugated secondary antibody at room temperature for 1 h. Membranes were developed using the Chemiluminescent HRP antibody detection reagent (Denville Scientific Inc., USA), and exposed to Kodak autoradiography films. Optical density of the bands was quantified using Quantity-one software (BIO-RAD, USA). To control for equal protein loading, expression of the proteins of interest was normalized to the β-actin or β-tubulin signal.

#### Immunohistochemistry

Using immunohistochemistry, the expression HIF-1α, VEGF-A and CD31 was assessed in mammary tumors of OID and control offspring. Briefly, tissues were fixed in 10% buffered formalin, embedded in paraffin, and sectioned (5 μm). Sections were deparaffinized in xylene, hydrated through graded alcohols, and incubated with H_2_O_2_ to block endogenous peroxidases. Antigen retrieval was carried out in a Target Retrieval solution (pH = 9; Dako S2368) in a pressure cooker for 20 minutes followed by 2 hours of cool down. Tissue sections were incubated with the specific primary antibody (see Table S6 for antibody specifications and dilutions used). After several washes, sections were treated with secondary antibody (anti-rabbit) and developed using the Dako EnVision+ Dual Link System-HRP, DAB^+^ (K4065) as instructed by the manufacturer. The sections were photographed using an Olympus BX61 DSU microscope at 60X magnification. Protein status of each sample was evaluated according to a modified version of the scoring system proposed by Allred and colleagues^65^. The total score for each sample was calculated as the sum of the estimated proportion of positive-staining cells (0–7) plus the estimated intensity of positive-staining cells (0–4).

### Collagen Staining

Staining was performed on 5 μm thick paraffin-embedded tumor sections with Sirius Red Staining. Briefly, after de-waxing and hydration, tumor sections were stained with picro-sirius red solution (0.5 g of Sirius red F3B (C.I. 35782) in 500 ml of saturated aqueous solution of picric acid) for 1 hour. Sections were washed in two changes of acidified water, dehydrated in three changes of 100% ethanol, cleared in xylene and, then, mounted in a resinous medium. The sections were photographed using an Olympus BX61 DSU microscope at 40X magnification. Staining was quantified by converting the image to gray scale and then isolating the red-stained collagen using thresholding and subsequent measure of the thresholded area with ImageJ software.

## Additional Information

**How to cite this article**: Fontelles, C. C. *et al*. Paternal overweight is associated with increased breast cancer risk in daughters in a mouse model. *Sci. Rep.* **6**, 28602; doi: 10.1038/srep28602 (2016).

## Supplementary Material

Supplementary Information

## Figures and Tables

**Table 1:** Top ten signaling pathways regulated by the microRNAs differentially expressed in OID fathers and their daughters.

Top ten signaling pathways regulated by miRNAs 296-5p, 874 and 1896
1. Insulin Receptor Signaling
2. Hypoxia Signaling
3. Regulation of the Epithelial-Mesenchymal Transition Pathway
4. Axonal Guidance Signaling
5. NANOG Pathway (Mammalian Embryonic Stem Cell Pluripotency)
6. SAPK/JNK Signaling
7. CDK5 Signaling
8. April Mediated Signaling
9. ERK/MAPK Signaling
10. Estrogen Receptor Signaling

Ingenuity Canonical Pathways regulated by mmu-miRNAs 296-5p, 874 and 1896.
