# Supplementary material for: Paternal overweight is associated with increased breast cancer risk in daughters in a mouse model
Source: Sci Rep. 2016 Jun 24;6:28602. doi: 10.1038/srep28602 (PMC4919621; doi:10.1038/srep28602)
Supplement: Supplementary Information [file srep28602-s1.pdf]

SUPPLEMENTARY INFORMATION FOR:

Paternal overweight is associated with increased breast cancer risk in daughters in a mouse model

Camile Castilho Fontelles[1,2], Elissa Carney[1], Johan Clarke[1], Nguyen Nguyen[1], Chao Yin[1], Lu Jin[1], M. Idalia Cruz[1], Thomas Prates Ong[2], Leena Hilakivi-Clarke[1] and Sonia de Assis[1]*

[1]Department of Oncology, Lombardi Comprehensive Cancer Center, Georgetown University, Washington, DC, USA

[2]Departament of Food and Experimental Nutrition, Faculty of Pharmaceutical Sciences, University of São Paulo and Food Research Center , São Paulo, SP, Brazil.

***Corresponding author:**

Sonia de Assis, Ph.D., 3970 Reservoir Road, NW, The Research Building, Room E410, Washington, DC 20057, USA

Phone: (202) 687-9518, Fax: (202) 687-7505, Email: deassiss@georgetown.edu

1. **Supplementary Figures and Tables**

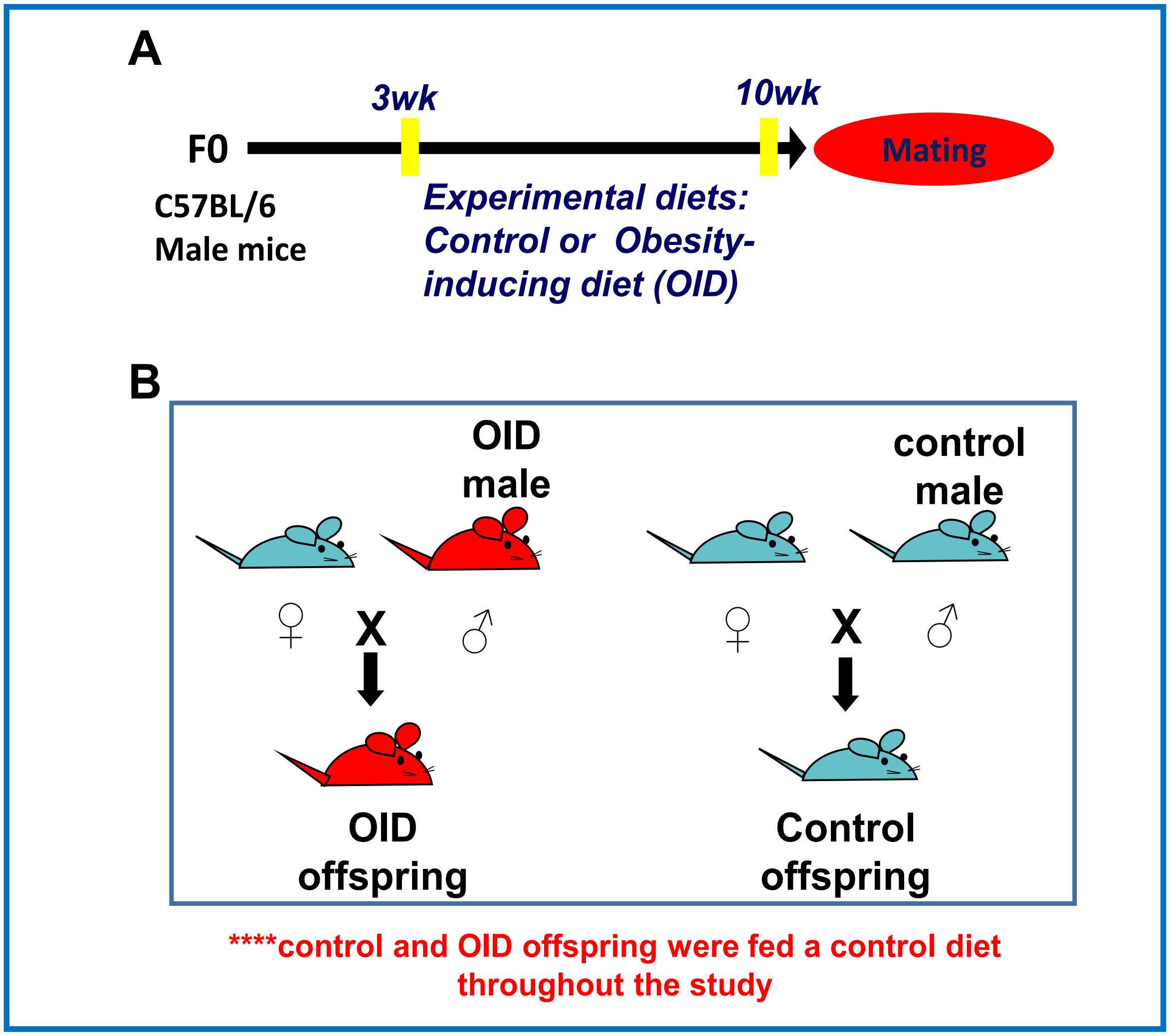

Figure S1.Experimental Design: (A) Male mice were fed the experimental diets (control or Obesity-inducing diet, OID) from 3 to 10 weeks of age. (B) At this point, control and OID-fed males were mated to control-fed females to generate the offspring as shown.

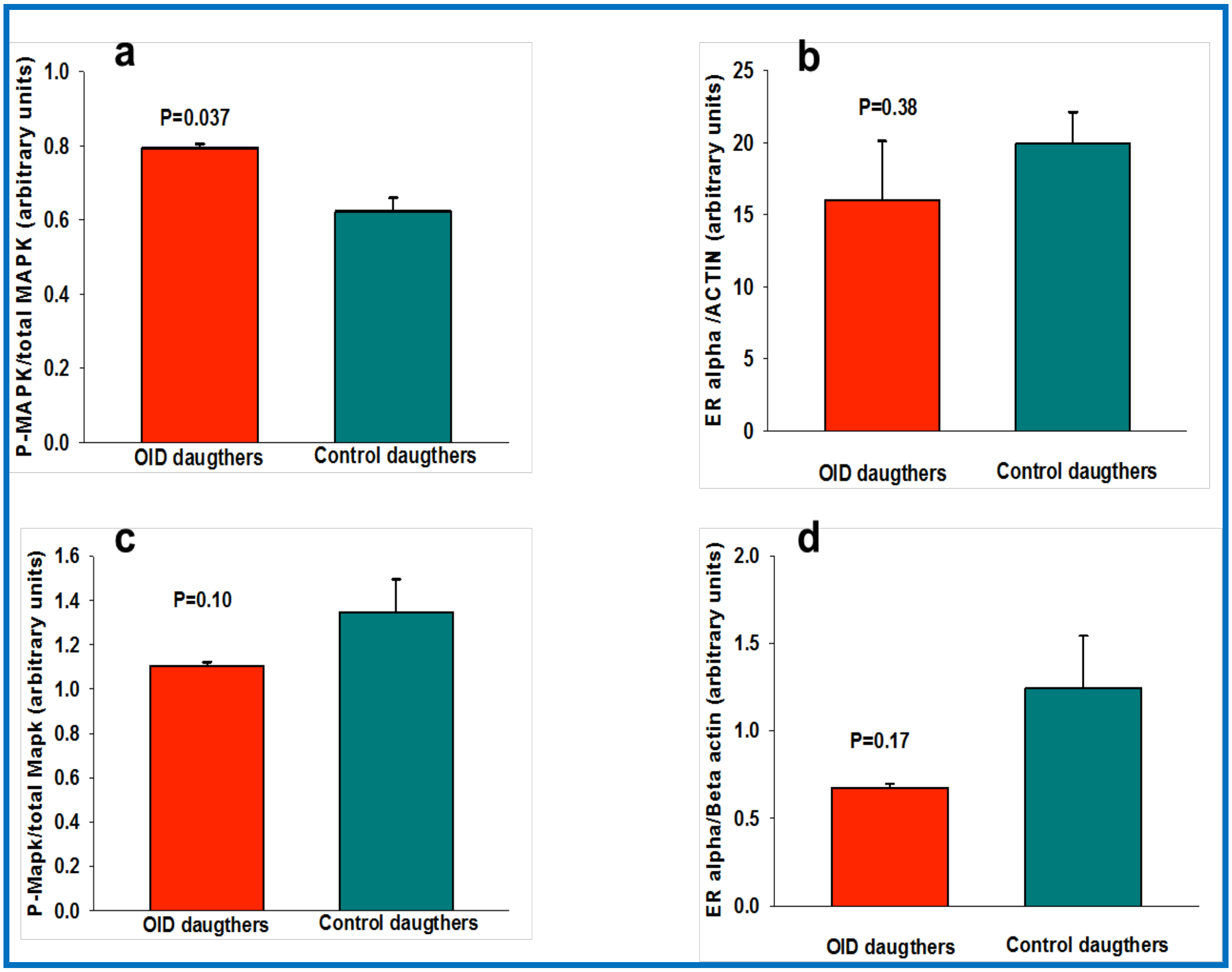

Figure S2. a-d, Quantification of MAPK and ER-α expression on PND50 in mammary tissue (**a**,**b**) and mammary tumors (**c**,**d**) of control and OID offspring. All data are mean± s.e.m. Significant differences versus the control group by t-test. *P*<0.05 is considered significant; exact *P* values are shown in each plot.

Control Group	Base-line weight	Final weight	Net weight gain
1	8.5	23	14.5
2	8.7	28.1	19.4
3	10.2	25.2	15
4	8.9	24.3	15.4
5	10.6	27	16.4
6	9.8	26.5	16.7
7	9.8	27.9	18.1
8	7.4	24	16.6
9	10	27.8	17.8
10	9.4	25.5	16.1
11	7.9	25.7	17.8
Mean	**9.2**	**25.9**	**16.7**
**OID Group**	**Base-line weight**	**Final weight**	**Net weight gain**
1	9.6	30.7	21.1
2	9	29.6	20.6
3	8.3	26.9	18.6
4	9.6	33.3	23.7
5	7.9	30.5	22.6
6	9.8	30.2	20.4
7	10.4	34.8	24.4
8	8.8	29.6	20.8
9	10.5	30.5	20
10	10.1	28.5	18.4
11	9.6	32.7	23.1
Mean	**9.4**	**30.7**	**21.2**

Table S1. **Paternal body weight** at base-line (3 weeks of age) and before mating (10 weeks of age) in OID (n=11) and control (n=11) groups.

**miRNA**	**Fold-difference**
mmu-miR-146b#-002453	**9.13**
mmu-miR-29a-002112	**-158.31**
mmu-miR-345-001137	**-2.81**
mmu-miR-1938-121194_mat	**-4.66**
mmu-miR-190-000489	**-2.59**
mmu-miR-761-002030	**-8.58**
mmu-miR-874-002268	**-4.24**
mmu-miR-205-000509	**-1.89**
mmu-miR-1896-121128_mat	**-4.79**
mmu-miR-345-5p-002528	**-1.73**
mmu-miR-296-5p-000527	**-3.50**

Table S2. List of microRNAs differentially expressed in OID fathers' sperm. miRNA that has fdr < 0.1 (p<0.05) were considered as significantly regulated miRNAs.

Control group	Number of TEBs
1	**10**
2	**10**
3	**6**
4	**7**
5	**12**
6	**11**
**Mean**	**9.3**
**SD**	**2.3**
**OID group**	**Number of TEBs**
1	**11**
2	**16**
3	**20**
4	**12**
5	**16**
**Mean**	**15***
**SD**	**3.6**

Table S3. Number of terminal end buds (TEBs, inguinal mammary gland) per animal in control and OID daughters. Significant differences versus the control group tested by t-test. *P<0.05 is considered significant; SD, Standard Deviation.

miRNA	Fold-difference
mmu-miR-327-002481	**-4.71**
mmu-miR-677-001660	**7.32**
mmu-miR-376a-001069	**-2.45**
mmu-miR-1839-5p-121135_mat	**-1.91**
mmu-miR-375-000564	**1.97**
mmu-miR-872-002264	**-1.15**
rno-miR-148b-5p-002058	**-3.43**
mmu-miR-98-000577	**-248641.51**
mmu-miR-692-001679	**-2.85**
mmu-miR-1897-5p-121199_mat	**-2480.46**
mmu-miR-599-241117_mat	**10.95**
mmu-miR-10b-001181	**-1.41**

mmu-miR-685-001670	**-2.11**
mmu-miR-218-1#-002552	**-1.67**
mmu-miR-701-001635	**-2.18**
rno-miR-327-001328	**-848.32**
mmu-miR-127-000452	**-1.98**
mmu-miR-874-002268	**-24.02**
mmu-miR-337-3p-002532	**-2.15**
mmu-let-7g-002282	**-1.59**
mmu-miR-1896-121128_mat	**-1.47**
hsa-miR-671-5p-197646_mat	**-1.49**
hsa-miR-183#-002270	**1.53**
mmu-miR-338-3p-002252	**-1.70**
mmu-miR-296-5p-000527	**-2.17**
mmu-miR-217-001133	**6.24**
mmu-miR-487b-001306	**-1.66**
mmu-miR-21#-002493	**-4.00**
hsa-miR-493-3p-001282	**-7.74**
mmu-miR-365-001020	**-1.93**
mmu-miR-7b-002555	**-5.39**
mmu-miR-467a-002587	**-1.71**
rno-miR-224-000599	**-1.97**
hsa-miR-214-000517	**-1.42**
mmu-miR-135a-000460	**-1.43**
mmu-miR-340-5p-002258	**-1.39**
rno-miR-664-001323	**-1.63**
mmu-miR-18a-002422	**-1.21**
hsa-miR-338-5P-002658	**-4.53**

Table S4. List of microRNAs differentially expressed in OID daughters' mammary tissue. miRNA that has fdr < 0.1 ($p<0.05$) were considered as significantly regulated miRNAs.

Ingenuity Canonical Pathways	-log(p value)	Ratio	Overlaps with dataset
Insulin Receptor Signaling	3.04E00	8.05E-02	12/149 (8%)
Hypoxia Signaling in the Cardiovascular System	2.99E00	1.18E-01	8/68 (12%)
Regulation of the Epithelial-Mesenchymal Transition Pathway	2.77E00	7.14E-02	14/196 (7%)
Axonal Guidance Signaling	2.59E00	5.13E-02	25/487 (5%)
Role of NANOG in Mammalian Embryonic Stem Cell Pluripotency	2.57E00	8.4E-02	10/119 (8%)
SAPK/JNK Signaling	2.54E00	8.57E-02	9/105 (9%)
CDK5 Signaling	2.2E00	8.25E-02	8/97 (8%)
April Mediated Signaling	2.2E00	1.14E-01	5/44 (11%)
ERK/MAPK Signaling	2.2E00	6.16E-02	13/211 (6%)
Estrogen Receptor Signaling	2.18E00	7.35E-02	10/136 (7%)
PEDF Signaling	2.17E00	8.86E-02	7/79 (9%)
Wnt/β-catenin Signaling	2.12E00	6.86E-02	12/175 (7%)
Basal Cell Carcinoma Signaling	2.11E00	8.97E-02	7/78 (9%)
STAT3 Pathway	2.11E00	8.75E-02	7/80 (9%)
B Cell Activating Factor Signaling	2.1E00	1.09E-01	5/46 (11%)
Breast Cancer Regulation by Stathmin1	2.1E00	6.07E-02	13/214 (6%)
Glucocorticoid Receptor Signaling	2.08E00	5.35E-02	16/299 (5%)
Ovarian Cancer Signaling	2.04E00	6.58E-02	10/152 (7%)
Mouse Embryonic Stem Cell Pluripotency	2.01E00	8.08E-02	8/99 (8%)
Cardiac β-adrenergic Signaling	2E00	6.33E-02	10/158 (6%)
PDGF Signaling	1.99E00	8.14E-02	7/86 (8%)
T Cell Receptor Signaling	1.96E00	7.34E-02	8/109 (7%)
Molecular Mechanisms of Cancer	1.93E00	4.9E-02	19/388 (5%)
Reelin Signaling in Neurons	1.93E00	8.24E-02	7/85 (8%)
Regulation of IL-2 Expression in Activated and Anergic T Lymphocytes	1.93E00	7.87E-02	7/89 (8%)
Cyclins and Cell Cycle Regulation	1.9E00	7.29E-02	7/96 (7%)
Sonic Hedgehog Signaling	1.87E00	1.14E-01	4/35 (11%)
Protein Ubiquitination Pathway	1.83E00	5.56E-02	15/270 (6%)
RAN Signaling	1.83E00	1.25E-01	3/24 (13%)
Cell Cycle: G1/S Checkpoint Regulation	1.77E00	8.33E-02	6/72 (8%)
Phosphatidylcholine Biosynthesis I	1.73E00	1.18E-01	2/17 (12%)
Oncostatin M Signaling	1.68E00	1.14E-01	4/35 (11%)
RhoGDI Signaling	1.67E00	5.45E-02	11/202 (5%)
Factors Promoting Cardiogenesis in Vertebrates	1.67E00	7.07E-02	7/99 (7%)
Ephrin Receptor Signaling	1.66E00	5.24E-02	11/210 (5%)
Renal Cell Carcinoma Signaling	1.65E00	7.59E-02	6/79 (8%)

Prolactin Signaling	1.57E00	7.14E-02	6/84 (7%)
B Cell Receptor Signaling	1.55E00	5.71E-02	10/175 (6%)
IL-4 Signaling	1.55E00	7.5E-02	6/80 (8%)
Signaling by Rho Family GTPases	1.52E00	4.94E-02	13/263 (5%)
Dopamine Receptor Signaling	1.5E00	6.25E-02	6/96 (6%)
BMP signaling pathway	1.5E00	6.98E-02	6/86 (7%)
Gα12/13 Signaling	1.49E00	6.3E-02	8/127 (6%)
D-myo-inositol-5-phosphate Metabolism	1.49E00	5.56E-02	9/162 (6%)
3-phosphoinositide Degradation	1.49E00	5.56E-02	9/162 (6%)
IGF-1 Signaling	1.49E00	6.54E-02	7/107 (7%)
ErbB2-ErbB3 Signaling	1.49E00	7.94E-02	5/63 (8%)
Netrin Signaling	1.48E00	6.9E-02	4/58 (7%)
Transcriptional Regulatory Network in Embryonic Stem Cells	1.45E00	9.3E-02	4/43 (9%)
Role of Wnt/GSK-3β Signaling in the Pathogenesis of Influenza	1.43E00	7.23E-02	6/83 (7%)
Estrogen-mediated S-phase Entry	1.42E00	1.07E-01	3/28 (11%)
Telomerase Signaling	1.41E00	6.6E-02	7/106 (7%)
D-myo-inositol (1,4,5,6)-Tetrakisphosphate Biosynthesis	1.4E00	5.56E-02	8/144 (6%)
D-myo-inositol (3,4,5,6)-tetrakisphosphate Biosynthesis	1.4E00	5.56E-02	8/144 (6%)
Colorectal Cancer Metastasis Signaling	1.38E00	4.85E-02	13/268 (5%)
Glioblastoma Multiforme Signaling	1.38E00	5.36E-02	9/168 (5%)
Rac Signaling	1.35E00	5.47E-02	7/128 (5%)
Antiproliferative Role of Somatostatin Receptor 2	1.3E00	6.94E-02	5/72 (7%)
fMLP Signaling in Neutrophils	1.26E00	5.3E-02	7/132 (5%)
TGF-β Signaling	1.26E00	6.38E-02	6/94 (6%)
PAK Signaling	1.24E00	5.45E-02	6/110 (5%)
JAK/Stat Signaling	1.23E00	7.04E-02	5/71 (7%)
Role of NFAT in Cardiac Hypertrophy	1.22E00	4.78E-02	10/209 (5%)
Choline Biosynthesis III	1.22E00	9.09E-02	2/22 (9%)
Neurotrophin/TRK Signaling	1.21E00	6.58E-02	5/76 (7%)
Ephrin A Signaling	1.2E00	7.41E-02	4/54 (7%)
Vitamin-C Transport	1.16E00	9.09E-02	2/22 (9%)
PKCθ Signaling in T Lymphocytes	1.14E00	4.86E-02	7/144 (5%)
IL-6 Signaling	1.14E00	5.65E-02	7/124 (6%)
Chronic Myeloid Leukemia Signaling	1.13E00	5.66E-02	6/106 (6%)
PTEN Signaling	1.13E00	5.07E-02	7/138 (5%)
IL-17 Signaling	1.13E00	6.67E-02	5/75 (7%)
LPS-stimulated MAPK Signaling	1.11E00	6.02E-02	5/83 (6%)
CNTF Signaling	1.1E00	7.02E-02	4/57 (7%)

Endometrial Cancer Signaling	1.1E00	6.67E-02	4/60 (7%)
IL-2 Signaling	1.08E00	6.56E-02	4/61 (7%)
p53 Signaling	1.06E00	5.31E-02	6/113 (5%)
Epithelial Adherens Junction Signaling	1.05E00	5.19E-02	8/154 (5%)
Gαi Signaling	1.05E00	5.19E-02	7/135 (5%)
G Protein Signaling Mediated by Tubby	1.05E00	6.82E-02	3/44 (7%)
Interferon Signaling	1.05E00	8.33E-02	3/36 (8%)
Diphthamide Biosynthesis	1.04E00	7.69E-02	1/13 (8%)
Thyronamine and Iodothyronamine Metabolism	1.04E00	7.69E-02	1/13 (8%)
Thyroid Hormone Metabolism I (via Deiodination)	1.04E00	7.69E-02	1/13 (8%)
S-adenosyl-L-methionine Biosynthesis	1.04E00	1.25E-01	1/8 (13%)
Acute Myeloid Leukemia Signaling	1.03E00	5.95E-02	5/84 (6%)
HGF Signaling	1.03E00	5.41E-02	6/111 (5%)
NF-κB Signaling	1.03E00	4.97E-02	9/181 (5%)
Cell Cycle Regulation by BTG Family Proteins	1.02E00	7.69E-02	3/39 (8%)
Cardiac Hypertrophy Signaling	1.01E00	4.4E-02	11/250 (4%)
Cholecystokinin/Gastrin-mediated Signaling	1.01E00	5.66E-02	6/106 (6%)
Paxillin Signaling	1.01E00	5.13E-02	6/117 (5%)
Glutathione Redox Reactions I	1.01E00	8.33E-02	2/24 (8%)
EGF Signaling	1.01E00	6.25E-02	4/64 (6%)
Role of Macrophages, Fibroblasts and Endothelial Cells in Rheumatoid Arthritis	1E00	4.09E-02	14/342 (4%)
HIF1α Signaling	9.82E-01	5.36E-02	6/112 (5%)
PPARα/RXRα Activation	9.8E-01	4.5E-02	9/200 (5%)
Sertoli Cell-Sertoli Cell Junction Signaling	9.8E-01	4.55E-02	9/198 (5%)
3-phosphoinositide Biosynthesis	9.79E-01	4.42E-02	8/181 (4%)
PI3K Signaling in B Lymphocytes	9.7E-01	4.9E-02	7/143 (5%)
G-Protein Coupled Receptor Signaling	9.49E-01	4.35E-02	12/276 (4%)
Cardiomyocyte Differentiation via BMP Receptors	9.29E-01	9.09E-02	2/22 (9%)
Phospholipase C Signaling	9.27E-01	4.15E-02	11/265 (4%)
NGF Signaling	9.24E-01	4.92E-02	6/122 (5%)
1,25-dihydroxyvitamin D3 Biosynthesis	9.23E-01	9.09E-02	1/11 (9%)
IL-15 Signaling	9.08E-01	5.56E-02	4/72 (6%)
Renin-Angiotensin Signaling	8.96E-01	4.76E-02	6/126 (5%)
TR/RXR Activation	8.94E-01	4.59E-02	5/109 (5%)
ErbB Signaling	8.94E-01	5.56E-02	5/90 (6%)

Role of JAK1 and JAK3 in γc Cytokine Signaling	8.89E-01	5.88E-02	4/68 (6%)
Thyroid Cancer Signaling	8.89E-01	6.82E-02	3/44 (7%)
Human Embryonic Stem Cell Pluripotency	8.81E-01	4.32E-02	7/162 (4%)
Estrogen-Dependent Breast Cancer Signaling	8.71E-01	5.48E-02	4/73 (5%)
GM-CSF Signaling	8.71E-01	5.88E-02	4/68 (6%)
Actin Cytoskeleton Signaling	8.68E-01	4.13E-02	10/242 (4%)
FcγRIIB Signaling in B Lymphocytes	8.66E-01	4.69E-02	3/64 (5%)
α-Adrenergic Signaling	8.49E-01	4.59E-02	5/109 (5%)
Superpathway of Inositol Phosphate Compounds	8.45E-01	3.85E-02	9/234 (4%)
Melanoma Signaling	8.43E-01	6E-02	3/50 (6%)
UVC-Induced MAPK Signaling	8.43E-01	6.82E-02	3/44 (7%)
Non-Small Cell Lung Cancer Signaling	8.36E-01	4.82E-02	4/83 (5%)
Protein Citrullination	8.33E-01	1.67E-01	1/6 (17%)
Galactose Degradation I (Leloir Pathway)	8.33E-01	6.25E-02	1/16 (6%)
UVA-Induced MAPK Signaling	8.06E-01	5.1E-02	5/98 (5%)
14-3-3-mediated Signaling	7.94E-01	4.96E-02	6/121 (5%)
Maturity Onset Diabetes of Young (MODY) Signaling	7.93E-01	6.06E-02	2/33 (6%)
VEGF Signaling	7.92E-01	4.59E-02	5/109 (5%)
GDNF Family Ligand-Receptor Interactions	7.86E-01	5.26E-02	4/76 (5%)
RhoA Signaling	7.82E-01	4.88E-02	6/123 (5%)
p38 MAPK Signaling	7.82E-01	5E-02	6/120 (5%)
Integrin Signaling	7.8E-01	4.33E-02	9/208 (4%)
Growth Hormone Signaling	7.7E-01	5.13E-02	4/78 (5%)
IL-22 Signaling	7.64E-01	8E-02	2/25 (8%)
Glycine Cleavage Complex	7.6E-01	6.67E-02	1/15 (7%)
Selenocysteine Biosynthesis II (Archaea and Eukaryotes)	7.6E-01	5.88E-02	1/17 (6%)
Glioma Signaling	7.52E-01	4.42E-02	5/113 (4%)
IL-3 Signaling	7.39E-01	5.33E-02	4/75 (5%)
Small Cell Lung Cancer Signaling	7.39E-01	4.26E-02	4/94 (4%)
Gαq Signaling	7.39E-01	4.09E-02	7/171 (4%)
Role of JAK family kinases in IL-6-type Cytokine Signaling	7.36E-01	7.14E-02	2/28 (7%)
Bupropion Degradation	7.1E-01	6.06E-02	2/33 (6%)
CXCR4 Signaling	6.9E-01	4.02E-02	7/174 (4%)
Acetone Degradation I (to Methylglyoxal)	6.85E-01	5.56E-02	2/36 (6%)
FLT3 Signaling in Hematopoietic Progenitor Cells	6.82E-01	5.06E-02	4/79 (5%)

Amyloid Processing	6.7E-01	4.92E-02	3/61 (5%)
Protein Kinase A Signaling	6.59E-01	3.67E-02	15/409 (4%)
Glycoaminoglycan-protein Linkage Region Biosynthesis	6.48E-01	6.25E-02	1/16 (6%)
eNOS Signaling	6.42E-01	3.87E-02	6/155 (4%)
Ceramide Signaling	6.17E-01	4.4E-02	4/91 (4%)
Role of p14/p19ARF in Tumor Suppression	6.17E-01	5.71E-02	2/35 (6%)
Pancreatic Adenocarcinoma Signaling	6.13E-01	3.91E-02	5/128 (4%)
Thrombopoietin Signaling	6.07E-01	4.69E-02	3/64 (5%)
Role of CHK Proteins in Cell Cycle Checkpoint Control	6.07E-01	5.08E-02	3/59 (5%)
AMPK Signaling	6.05E-01	3.31E-02	6/181 (3%)
Prostanoid Biosynthesis	6.04E-01	6.67E-02	1/15 (7%)
Thrombin Signaling	6.02E-01	3.79E-02	8/211 (4%)
mTOR Signaling	6.02E-01	3.76E-02	8/213 (4%)
Role of Osteoblasts, Osteoclasts and Chondrocytes in Rheumatoid Arthritis	5.97E-01	3.6E-02	9/250 (4%)
Toll-like Receptor Signaling	5.93E-01	4.69E-02	3/64 (5%)
Fc Epsilon RI Signaling	5.92E-01	4.27E-02	5/117 (4%)
Prostate Cancer Signaling	5.82E-01	3.88E-02	4/103 (4%)
Huntington's Disease Signaling	5.76E-01	3.57E-02	9/252 (4%)
Embryonic Stem Cell Differentiation into Cardiac Lineages	5.65E-01	1E-01	1/10 (10%)
Calcium Transport I	5.65E-01	7.14E-02	1/14 (7%)
Myc Mediated Apoptosis Signaling	5.65E-01	4.76E-02	3/63 (5%)
Death Receptor Signaling	5.65E-01	4.41E-02	3/68 (4%)
Glutamate Receptor Signaling	5.51E-01	4.17E-02	3/72 (4%)
Circadian Rhythm Signaling	5.4E-01	5.26E-02	2/38 (5%)
Role of NFAT in Regulation of the Immune Response	5.39E-01	3.5E-02	7/200 (4%)
Role of BRCA1 in DNA Damage Response	5.38E-01	4.23E-02	3/71 (4%)
Apoptosis Signaling	5.27E-01	4E-02	4/100 (4%)
Type II Diabetes Mellitus Signaling	5.26E-01	2.92E-02	5/171 (3%)
Hereditary Breast Cancer Signaling	5.26E-01	3.73E-02	5/134 (4%)
tRNA Splicing	5.23E-01	4.35E-02	2/46 (4%)
Bladder Cancer Signaling	5.17E-01	4.12E-02	4/97 (4%)
G Beta Gamma Signaling	5.17E-01	3.31E-02	4/121 (3%)
CD40 Signaling	5.01E-01	4.23E-02	3/71 (4%)
Cleavage and Polyadenylation of Pre-mRNA	4.99E-01	7.69E-02	1/13 (8%)
Synaptic Long Term Potentiation	4.91E-01	3.85E-02	5/130 (4%)
ERK5 Signaling	4.89E-01	4.41E-02	3/68 (4%)
IL-17A Signaling in Airway Cells	4.89E-01	3.95E-02	3/76 (4%)

HMGB1 Signaling	4.78E-01	3.67E-02	4/109 (4%)
NRF2-mediated Oxidative Stress Response	4.76E-01	3.59E-02	7/195 (4%)
Calcium Signaling	4.76E-01	3.23E-02	7/217 (3%)
Estrogen Biosynthesis	4.75E-01	4.08E-02	2/49 (4%)
Assembly of RNA Polymerase III Complex	4.7E-01	6.25E-02	1/16 (6%)
Cholesterol Biosynthesis I	4.7E-01	2.5E-02	1/40 (3%)
Cholesterol Biosynthesis II (via 24,25-dihydrolanosterol)	4.7E-01	2.5E-02	1/40 (3%)
Cholesterol Biosynthesis III (via Desmosterol)	4.7E-01	2.5E-02	1/40 (3%)
PPAR Signaling	4.69E-01	3.74E-02	4/107 (4%)
PI3K/AKT Signaling	4.67E-01	3.29E-02	5/152 (3%)
PXR/RXR Activation	4.56E-01	3.26E-02	3/92 (3%)
Erythropoietin Signaling	4.56E-01	3.8E-02	3/79 (4%)
Retinoic acid Mediated Apoptosis Signaling	4.56E-01	4.11E-02	3/73 (4%)
EIF2 Signaling	4.51E-01	3.48E-02	7/201 (3%)
Role of PKR in Interferon Induction and Antiviral Response	4.47E-01	4.08E-02	2/49 (4%)
Neuroprotective Role of THOP1 in Alzheimer's Disease	4.47E-01	3.64E-02	2/55 (4%)
IL-10 Signaling	4.45E-01	3.85E-02	3/78 (4%)
Agrin Interactions at Neuromuscular Junction	4.45E-01	4.29E-02	3/70 (4%)
Role of MAPK Signaling in the Pathogenesis of Influenza	4.45E-01	4.17E-02	3/72 (4%)
Macropinocytosis Signaling	4.45E-01	3.9E-02	3/77 (4%)
Colanic Acid Building Blocks Biosynthesis	4.44E-01	2.78E-02	1/36 (3%)
Melatonin Signaling	4.25E-01	3.7E-02	3/81 (4%)
Cellular Effects of Sildenafil (Viagra)	4.22E-01	3.23E-02	5/155 (3%)
Telomere Extension by Telomerase	4.21E-01	5.56E-02	1/18 (6%)
CDP-diacylglycerol Biosynthesis I	4.21E-01	3.7E-02	1/27 (4%)
γ-glutamyl Cycle	4.21E-01	3.57E-02	1/28 (4%)
GNRH Signaling	4.15E-01	3.27E-02	5/153 (3%)
Nitric Oxide Signaling in the Cardiovascular System	4.1E-01	3.2E-02	4/125 (3%)
Methionine Degradation I (to Homocysteine)	3.99E-01	4.17E-02	1/24 (4%)
Dopamine-DARPP32 Feedback in cAMP Signaling	3.96E-01	3.21E-02	6/187 (3%)
iNOS Signaling	3.95E-01	3.77E-02	2/53 (4%)
Ephrin B Signaling	3.88E-01	3.66E-02	3/82 (4%)
Phosphatidylglycerol Biosynthesis II (Non-plastidic)	3.79E-01	3.03E-02	1/33 (3%)
Leptin Signaling in Obesity	3.79E-01	3.53E-02	3/85 (4%)

HER-2 Signaling in Breast Cancer	3.7E-01	3.66E-02	3/82 (4%)
VEGF Family Ligand-Receptor Interactions	3.7E-01	3.41E-02	3/88 (3%)
Relaxin Signaling	3.68E-01	3.05E-02	5/164 (3%)
iCOS-iCOSL Signaling in T Helper Cells	3.65E-01	3.17E-02	4/126 (3%)
Acute Phase Response Signaling	3.61E-01	3.31E-02	6/181 (3%)
D-myo-inositol (1,4,5)-trisphosphate Degradation	3.61E-01	4.35E-02	1/23 (4%)
Cysteine Biosynthesis III (mammalia)	3.61E-01	3.23E-02	1/31 (3%)
Endoplasmic Reticulum Stress Pathway	3.61E-01	5.26E-02	1/19 (5%)
Type I Diabetes Mellitus Signaling	3.58E-01	3.31E-02	4/121 (3%)
Hepatic Cholestasis	3.56E-01	2.73E-02	5/183 (3%)
Natural Killer Cell Signaling	3.44E-01	3.39E-02	4/118 (3%)
Granzyme A Signaling	3.43E-01	5E-02	1/20 (5%)
Role of Tissue Factor in Cancer	3.38E-01	3.08E-02	4/130 (3%)
DNA Methylation and Transcriptional Repression Signaling	3.27E-01	4.35E-02	1/23 (4%)
Nicotine Degradation III	3.12E-01	2.74E-02	2/73 (3%)
Production of Nitric Oxide and Reactive Oxygen Species in Macrophages	3.09E-01	2.83E-02	6/212 (3%)
Regulation of eIF4 and p70S6K Signaling	3.05E-01	2.86E-02	5/175 (3%)
UVB-Induced MAPK Signaling	3.03E-01	3.45E-02	2/58 (3%)
Polyamine Regulation in Colon Cancer	2.98E-01	3.33E-02	1/30 (3%)
Lymphotoxin β Receptor Signaling	2.95E-01	3.23E-02	2/62 (3%)
Melanocyte Development and Pigmentation Signaling	2.95E-01	3.16E-02	3/95 (3%)
RANK Signaling in Osteoclasts	2.88E-01	3.09E-02	3/97 (3%)
Melatonin Degradation I	2.86E-01	3.03E-02	2/66 (3%)
p70S6K Signaling	2.84E-01	3.03E-02	4/132 (3%)
Neuregulin Signaling	2.82E-01	2.88E-02	3/104 (3%)
CTLA4 Signaling in Cytotoxic T Lymphocytes	2.82E-01	3.12E-02	3/96 (3%)
Aldosterone Signaling in Epithelial Cells	2.8E-01	2.96E-02	5/169 (3%)
Actin Nucleation by ARP-WASP Complex	2.78E-01	2.99E-02	2/67 (3%)
Virus Entry via Endocytic Pathways	2.75E-01	2.97E-02	3/101 (3%)
IL-8 Signaling	2.73E-01	2.67E-02	6/225 (3%)
ILK Signaling	2.73E-01	2.93E-02	6/205 (3%)
P2Y Purigenic Receptor Signaling Pathway	2.73E-01	2.78E-02	4/144 (3%)
Role of JAK1, JAK2 and TYK2 in Interferon Signaling	2.72E-01	3.57E-02	1/28 (4%)

Pyrimidine Deoxyribonucleotides De Novo Biosynthesis I	2.72E-01	2.27E-02	1/44 (2%)
Superpathway of D-myo-inositol (1,4,5)-trisphosphate Metabolism	2.72E-01	3.03E-02	1/33 (3%)
cAMP-mediated signaling	2.71E-01	3.1E-02	7/226 (3%)
Dermatan Sulfate Biosynthesis	2.7E-01	2.74E-02	2/73 (3%)
Regulation of Cellular Mechanics by Calpain Protease	2.7E-01	2.74E-02	2/73 (3%)
Germ Cell-Sertoli Cell Junction Signaling	2.66E-01	2.96E-02	5/169 (3%)
Gap Junction Signaling	2.66E-01	2.76E-02	5/181 (3%)
Glioma Invasiveness Signaling	2.63E-01	3.03E-02	2/66 (3%)
Heparan Sulfate Biosynthesis	2.63E-01	2.67E-02	2/75 (3%)
IL-17A Signaling in Gastric Cells	2.61E-01	3.57E-02	1/28 (4%)
Induction of Apoptosis by HIV1	2.56E-01	2.99E-02	2/67 (3%)
IL-1 Signaling	2.52E-01	2.75E-02	3/109 (3%)
Lipid Antigen Presentation by CD1	2.5E-01	3.33E-02	1/30 (3%)
ErbB4 Signaling	2.48E-01	2.9E-02	2/69 (3%)
Superpathway of Melatonin Degradation	2.48E-01	2.47E-02	2/81 (2%)
Nicotine Degradation II	2.42E-01	2.35E-02	2/85 (2%)
IL-15 Production	2.39E-01	3.23E-02	1/31 (3%)
TNFR2 Signaling	2.29E-01	2.94E-02	1/34 (3%)
Glutathione-mediated Detoxification	2.29E-01	2.27E-02	1/44 (2%)
Pyrimidine Ribonucleotides Interconversion	2.29E-01	2.5E-02	1/40 (3%)
Activation of IRF by Cytosolic Pattern Recognition Receptors	2.28E-01	2.7E-02	2/74 (3%)
Eicosanoid Signaling	2.28E-01	2.33E-02	2/86 (2%)
Pyrimidine Ribonucleotides De Novo Biosynthesis	2.11E-01	1.85E-02	1/54 (2%)
Superpathway of Cholesterol Biosynthesis	2.11E-01	1.15E-02	1/87 (1%)
4-1BB Signaling in T Lymphocytes	2.03E-01	2.78E-02	1/36 (3%)
Superpathway of Methionine Degradation	2.03E-01	1.54E-02	1/65 (2%)

Table S5. **Signaling pathways regulated by the microRNAs differentially expressed in OID fathers and their daughters.** Ingenuity Canonical Pathways regulated by mmu-miRNAs 296-5p, 874 and 1896.

Ingenuity Canonical Pathways	-log(p-value)	Ratio	Overlaps with dataset
Role of NANOG in Mammalian Embryonic Stem Cell Pluripotency	2E00	2.73E-02	3/110 (3%)
Thyroid Cancer Signaling	1.95E00	5E-02	2/40 (5%)
S-adenosyl-L-methionine Biosynthesis	1.92E00	3.33E-01	1/3 (33%)
Melanoma Signaling	1.91E00	4.76E-02	2/42 (5%)
Molecular Mechanisms of Cancer	1.81E00	1.38E-02	5/363 (1%)
Ovarian Cancer Signaling	1.8E00	2.29E-02	3/131 (2%)
CD27 Signaling in Lymphocytes	1.73E00	3.85E-02	2/52 (4%)
Galactose Degradation I (Leloir Pathway)	1.7E00	2E-01	1/5 (20%)
Lymphotoxin β Receptor Signaling	1.7E00	3.7E-02	2/54 (4%)
Glioblastoma Multiforme Signaling	1.67E00	2.05E-02	3/146 (2%)
Regulation of Cellular Mechanics by Calpain Protease	1.65E00	3.51E-02	2/57 (4%)
Myc Mediated Apoptosis Signaling	1.64E00	3.45E-02	2/58 (3%)
Non-Small Cell Lung Cancer Signaling	1.55E00	3.08E-02	2/65 (3%)
Small Cell Lung Cancer Signaling	1.48E00	2.82E-02	2/71 (3%)
IL-17 Signaling	1.47E00	2.78E-02	2/72 (3%)
Basal Cell Carcinoma Signaling	1.47E00	2.78E-02	2/72 (3%)
BMP signaling pathway	1.43E00	2.67E-02	2/75 (3%)
Ceramide Signaling	1.38E00	2.5E-02	2/80 (3%)
Bladder Cancer Signaling	1.32E00	2.3E-02	2/87 (2%)
TGF-β Signaling	1.32E00	2.3E-02	2/87 (2%)
G Beta Gamma Signaling	1.31E00	2.27E-02	2/88 (2%)
PAK Signaling	1.31E00	2.27E-02	2/88 (2%)
UVA-Induced MAPK Signaling	1.31E00	2.27E-02	2/88 (2%)
Apoptosis Signaling	1.3E00	2.25E-02	2/89 (2%)
Role of IL-17A in Psoriasis	1.29E00	7.69E-02	1/13 (8%)
Chronic Myeloid Leukemia Signaling	1.28E00	2.17E-02	2/92 (2%)
Colanic Acid Building Blocks Biosynthesis	1.26E00	7.14E-02	1/14 (7%)
SAPK/JNK Signaling	1.26E00	2.13E-02	2/94 (2%)
T Cell Receptor Signaling	1.23E00	2.06E-02	2/97 (2%)
p53 Signaling	1.23E00	2.04E-02	2/98 (2%)
Glioma Signaling	1.23E00	2.04E-02	2/98 (2%)
Cardiac Hypertrophy Signaling	1.21E00	1.35E-02	3/223 (1%)
Granzyme B Signaling	1.2E00	6.25E-02	1/16 (6%)
Methionine Degradation I (to Homocysteine)	1.2E00	6.25E-02	1/16 (6%)
Parkinson's Signaling	1.2E00	6.25E-02	1/16 (6%)
HGF Signaling	1.17E00	1.9E-02	2/105 (2%)
NGF Signaling	1.16E00	1.87E-02	2/107 (2%)
Differential Regulation of Cytokine Production in Macrophages and T	1.15E00	5.56E-02	1/18 (6%)

Helper Cells by IL-17A and IL-17F			
Valine Degradation I	1.15E00	5.56E-02	1/18 (6%)
Cysteine Biosynthesis III (mammalia)	1.15E00	5.56E-02	1/18 (6%)
Oxidative Phosphorylation	1.15E00	1.85E-02	2/108 (2%)
Role of Tissue Factor in Cancer	1.14E00	1.82E-02	2/110 (2%)
Cardiomyocyte Differentiation via BMP Receptors	1.13E00	5.26E-02	1/19 (5%)
GADD45 Signaling	1.13E00	5.26E-02	1/19 (5%)
PKCθ Signaling in T Lymphocytes	1.1E00	1.72E-02	2/116 (2%)
Gα12/13 Signaling	1.09E00	1.71E-02	2/117 (2%)
Gαi Signaling	1.07E00	1.67E-02	2/120 (2%)
Differential Regulation of Cytokine Production in Intestinal Epithelial Cells by IL-17A and IL-17F	1.05E00	4.35E-02	1/23 (4%)
Tumoricidal Function of Hepatic Natural Killer Cells	1.03E00	4.17E-02	1/24 (4%)
Estrogen-mediated S-phase Entry	1.03E00	4.17E-02	1/24 (4%)
GNRH Signaling	1.03E00	1.56E-02	2/128 (2%)
Hereditary Breast Cancer Signaling	1.03E00	1.56E-02	2/128 (2%)
Xenobiotic Metabolism Signaling	1.02E00	1.12E-02	3/269 (1%)
IL-17A Signaling in Gastric Cells	1.02E00	4E-02	1/25 (4%)
Axonal Guidance Signaling	1.01E00	9.24E-03	4/433 (1%)
Human Embryonic Stem Cell Pluripotency	9.99E-01	1.5E-02	2/133 (2%)
Cell Cycle Control of Chromosomal Replication	9.86E-01	3.7E-02	1/27 (4%)
Superpathway of Methionine Degradation	9.3E-01	3.23E-02	1/31 (3%)
Cytotoxic T Lymphocyte-mediated Apoptosis of Target Cells	9.17E-01	3.12E-02	1/32 (3%)
CXCR4 Signaling	9.08E-01	1.32E-02	2/151 (1%)
Circadian Rhythm Signaling	9.04E-01	3.03E-02	1/33 (3%)
Retinoate Biosynthesis I	9.04E-01	3.03E-02	1/33 (3%)
Oncostatin M Signaling	8.92E-01	2.94E-02	1/34 (3%)
TWEAK Signaling	8.92E-01	2.94E-02	1/34 (3%)
Cell Cycle Regulation by BTG Family Proteins	8.8E-01	2.86E-02	1/35 (3%)
Germ Cell-Sertoli Cell Junction Signaling	8.71E-01	1.26E-02	2/159 (1%)
Docosahexaenoic Acid (DHA) Signaling	8.37E-01	2.56E-02	1/39 (3%)
Acute Phase Response Signaling	8.29E-01	1.18E-02	2/169 (1%)
Mitochondrial Dysfunction	8.29E-01	1.18E-02	2/169 (1%)
Role of PKR in Interferon Induction and Antiviral Response	8.26E-01	2.5E-02	1/40 (3%)
Neuroprotective Role of THOP1 in Alzheimer's Disease	8.26E-01	2.5E-02	1/40 (3%)
NF-κB Signaling	8.21E-01	1.17E-02	2/171 (1%)
FcγRIIB Signaling in B Lymphocytes	8.17E-01	2.44E-02	1/41 (2%)
B Cell Receptor Signaling	8.09E-01	1.15E-02	2/174 (1%)
UVC-Induced MAPK Signaling	8.07E-01	2.38E-02	1/42 (2%)

Sertoli Cell-Sertoli Cell Junction Signaling	7.93E-01	1.12E-02	2/178 (1%)
Role of IL-17F in Allergic Inflammatory Airway Diseases	7.88E-01	2.27E-02	1/44 (2%)
IL-8 Signaling	7.75E-01	1.09E-02	2/183 (1%)
Regulation of the Epithelial-Mesenchymal Transition Pathway	7.71E-01	1.09E-02	2/184 (1%)
Hematopoiesis from Pluripotent Stem Cells	7.62E-01	2.13E-02	1/47 (2%)
Primary Immunodeficiency Signaling	7.54E-01	2.08E-02	1/48 (2%)
TNFR1 Signaling	7.54E-01	2.08E-02	1/48 (2%)
Thrombin Signaling	7.53E-01	1.06E-02	2/189 (1%)
CNTF Signaling	7.23E-01	1.92E-02	1/52 (2%)
Endometrial Cancer Signaling	7.23E-01	1.92E-02	1/52 (2%)
IL-2 Signaling	7.15E-01	1.89E-02	1/53 (2%)
Nur77 Signaling in T Lymphocytes	7.08E-01	1.85E-02	1/54 (2%)
Role of IL-17A in Arthritis	7.08E-01	1.85E-02	1/54 (2%)
Thrombopoietin Signaling	7.01E-01	1.82E-02	1/55 (2%)
Integrin Signaling	6.99E-01	9.76E-03	2/205 (1%)
Actin Nucleation by ARP-WASP Complex	6.94E-01	1.79E-02	1/56 (2%)
Glioma Invasiveness Signaling	6.87E-01	1.75E-02	1/57 (2%)
ErbB2-ErbB3 Signaling	6.87E-01	1.75E-02	1/57 (2%)
Actin Cytoskeleton Signaling	6.72E-01	9.35E-03	2/214 (1%)
Induction of Apoptosis by HIV1	6.67E-01	1.67E-02	1/60 (2%)
ErbB4 Signaling	6.67E-01	1.67E-02	1/60 (2%)
Retinoic acid Mediated Apoptosis Signaling	6.61E-01	1.64E-02	1/61 (2%)
Role of Osteoblasts, Osteoclasts and Chondrocytes in Rheumatoid Arthritis	6.6E-01	9.17E-03	2/218 (1%)
cAMP-mediated signaling	6.57E-01	9.13E-03	2/219 (1%)
Role of JAK1 and JAK3 in γc Cytokine Signaling	6.55E-01	1.61E-02	1/62 (2%)
GM-CSF Signaling	6.55E-01	1.61E-02	1/62 (2%)
IL-15 Signaling	6.48E-01	1.59E-02	1/63 (2%)
Antiproliferative Role of Somatostatin Receptor 2	6.48E-01	1.59E-02	1/63 (2%)
ERK5 Signaling	6.48E-01	1.59E-02	1/63 (2%)
Estrogen-Dependent Breast Cancer Signaling	6.48E-01	1.59E-02	1/63 (2%)
Pyridoxal 5'-phosphate Salvage Pathway	6.48E-01	1.59E-02	1/63 (2%)
PCP pathway	6.48E-01	1.59E-02	1/63 (2%)
Cell Cycle: G1/S Checkpoint Regulation	6.48E-01	1.59E-02	1/63 (2%)
IL-17A Signaling in Airway Cells	6.42E-01	1.56E-02	1/64 (2%)
Angiopoietin Signaling	6.37E-01	1.54E-02	1/65 (2%)
Hypoxia Signaling in the Cardiovascular System	6.37E-01	1.54E-02	1/65 (2%)
Erythropoietin Signaling	6.25E-01	1.49E-02	1/67 (1%)

Neurotrophin/TRK Signaling	6.25E-01	1.49E-02	1/67 (1%)
Agrin Interactions at Neuromuscular Junction	6.19E-01	1.47E-02	1/68 (1%)
Role of MAPK Signaling in the Pathogenesis of Influenza	6.19E-01	1.47E-02	1/68 (1%)
GDNF Family Ligand-Receptor Interactions	6.19E-01	1.47E-02	1/68 (1%)
Macropinocytosis Signaling	6.19E-01	1.47E-02	1/68 (1%)
Phospholipase C Signaling	6.12E-01	8.51E-03	2/235 (1%)
Colorectal Cancer Metastasis Signaling	6.1E-01	8.47E-03	2/236 (1%)
Renal Cell Carcinoma Signaling	6.08E-01	1.43E-02	1/70 (1%)
Chemokine Signaling	6.08E-01	1.43E-02	1/70 (1%)
IL-3 Signaling	6.03E-01	1.41E-02	1/71 (1%)
PEDF Signaling	6.03E-01	1.41E-02	1/71 (1%)
GPCR-Mediated Integration of Enteroendocrine Signaling Exemplified by an L Cell	6.03E-01	1.41E-02	1/71 (1%)
JAK/Stat Signaling	5.98E-01	1.39E-02	1/72 (1%)
LPS-stimulated MAPK Signaling	5.93E-01	1.37E-02	1/73 (1%)
NF-κB Activation by Viruses	5.93E-01	1.37E-02	1/73 (1%)
Prolactin Signaling	5.93E-01	1.37E-02	1/73 (1%)
STAT3 Pathway	5.93E-01	1.37E-02	1/73 (1%)
IL-4 Signaling	5.93E-01	1.37E-02	1/73 (1%)
FLT3 Signaling in Hematopoietic Progenitor Cells	5.88E-01	1.35E-02	1/74 (1%)
HER-2 Signaling in Breast Cancer	5.78E-01	1.32E-02	1/76 (1%)
Role of Wnt/GSK-3β Signaling in the Pathogenesis of Influenza	5.78E-01	1.32E-02	1/76 (1%)
VEGF Family Ligand-Receptor Interactions	5.78E-01	1.32E-02	1/76 (1%)
Cyclins and Cell Cycle Regulation	5.73E-01	1.3E-02	1/77 (1%)
PDGF Signaling	5.73E-01	1.3E-02	1/77 (1%)
VDR/RXR Activation	5.68E-01	1.28E-02	1/78 (1%)
Acute Myeloid Leukemia Signaling	5.63E-01	1.27E-02	1/79 (1%)
Regulation of IL-2 Expression in Activated and Anergic T Lymphocytes	5.63E-01	1.27E-02	1/79 (1%)
G-Protein Coupled Receptor Signaling	5.6E-01	7.81E-03	2/256 (1%)
Prostate Cancer Signaling	5.5E-01	1.22E-02	1/82 (1%)
Melanocyte Development and Pigmentation Signaling	5.41E-01	1.19E-02	1/84 (1%)
ErbB Signaling	5.37E-01	1.18E-02	1/85 (1%)
α-Adrenergic Signaling	5.32E-01	1.16E-02	1/86 (1%)
FAK Signaling	5.32E-01	1.16E-02	1/86 (1%)
Neuregulin Signaling	5.24E-01	1.14E-02	1/88 (1%)
CTLA4 Signaling in Cytotoxic T Lymphocytes	5.24E-01	1.14E-02	1/88 (1%)
RANK Signaling in Osteoclasts	5.24E-01	1.14E-02	1/88 (1%)
Regulation of Actin-based Motility	5.2E-01	1.12E-02	1/89 (1%)

by Rho			
Virus Entry via Endocytic Pathways	5.2E-01	1.12E-02	1/89 (1%)
Communication between Innate and Adaptive Immune Cells	5.2E-01	1.12E-02	1/89 (1%)
Glucocorticoid Receptor Signaling	5.17E-01	7.27E-03	2/275 (1%)
Factors Promoting Cardiogenesis in Vertebrates	5.12E-01	1.1E-02	1/91 (1%)
Salvage Pathways of Pyrimidine Ribonucleotides	5.12E-01	1.1E-02	1/91 (1%)
VEGF Signaling	5.12E-01	1.1E-02	1/91 (1%)
Death Receptor Signaling	5.08E-01	1.09E-02	1/92 (1%)
PPAR Signaling	5.04E-01	1.08E-02	1/93 (1%)
Mouse Embryonic Stem Cell Pluripotency	4.96E-01	1.05E-02	1/95 (1%)
IGF-1 Signaling	4.89E-01	1.03E-02	1/97 (1%)
Amyotrophic Lateral Sclerosis Signaling	4.85E-01	1.02E-02	1/98 (1%)
Telomerase Signaling	4.85E-01	1.02E-02	1/98 (1%)
CDK5 Signaling	4.82E-01	1.01E-02	1/99 (1%)
Paxillin Signaling	4.78E-01	1E-02	1/100 (1%)
Role of Macrophages, Fibroblasts and Endothelial Cells in Rheumatoid Arthritis	4.77E-01	6.78E-03	2/295 (1%)
HIF1α Signaling	4.75E-01	9.9E-03	1/101 (1%)
Cholecystokinin/Gastrin-mediated Signaling	4.75E-01	9.9E-03	1/101 (1%)
Rac Signaling	4.68E-01	9.71E-03	1/103 (1%)
Pancreatic Adenocarcinoma Signaling	4.58E-01	9.43E-03	1/106 (1%)
Fc Epsilon RI Signaling	4.51E-01	9.26E-03	1/108 (1%)
fMLP Signaling in Neutrophils	4.51E-01	9.26E-03	1/108 (1%)
Renin-Angiotensin Signaling	4.51E-01	9.26E-03	1/108 (1%)
Type I Diabetes Mellitus Signaling	4.51E-01	9.26E-03	1/108 (1%)
Natural Killer Cell Signaling	4.48E-01	9.17E-03	1/109 (1%)
CCR3 Signaling in Eosinophils	4.3E-01	8.7E-03	1/115 (1%)
IL-6 Signaling	4.27E-01	8.62E-03	1/116 (1%)
14-3-3-mediated Signaling	4.24E-01	8.55E-03	1/117 (1%)
PTEN Signaling	4.21E-01	8.47E-03	1/118 (1%)
Synaptic Long Term Potentiation	4.18E-01	8.4E-03	1/119 (1%)
HMGB1 Signaling	4.18E-01	8.4E-03	1/119 (1%)
p70S6K Signaling	4.18E-01	8.4E-03	1/119 (1%)
P2Y Purigenic Receptor Signaling Pathway	4.18E-01	8.4E-03	1/119 (1%)
RhoA Signaling	4.12E-01	8.26E-03	1/121 (1%)
PI3K/AKT Signaling	4.07E-01	8.13E-03	1/123 (1%)
D-myo-inositol (1,4,5,6)-Tetrakisphosphate Biosynthesis	3.99E-01	7.94E-03	1/126 (1%)
D-myo-inositol (3,4,5,6)-tetrakisphosphate Biosynthesis	3.99E-01	7.94E-03	1/126 (1%)
PI3K Signaling in B Lymphocytes	3.96E-01	7.87E-03	1/127 (1%)

Cellular Effects of Sildenafil (Viagra)	3.93E-01	7.81E-03	1/128 (1%)
Estrogen Receptor Signaling	3.93E-01	7.81E-03	1/128 (1%)
Insulin Receptor Signaling	3.83E-01	7.58E-03	1/132 (1%)
Adipogenesis pathway	3.81E-01	7.52E-03	1/133 (1%)
Cardiac β-adrenergic Signaling	3.81E-01	7.52E-03	1/133 (1%)
Aryl Hydrocarbon Receptor Signaling	3.66E-01	7.19E-03	1/139 (1%)
Synaptic Long Term Depression	3.62E-01	7.09E-03	1/141 (1%)
D-myo-inositol-5-phosphate Metabolism	3.59E-01	7.04E-03	1/142 (1%)
3-phosphoinositide Degradation	3.57E-01	6.99E-03	1/143 (1%)
Regulation of eIF4 and p70S6K Signaling	3.53E-01	6.9E-03	1/145 (1%)
Epithelial Adherens Junction Signaling	3.5E-01	6.85E-03	1/146 (1%)
Cdc42 Signaling	3.38E-01	6.58E-03	1/152 (1%)
3-phosphoinositide Biosynthesis	3.36E-01	6.54E-03	1/153 (1%)
Gap Junction Signaling	3.32E-01	6.45E-03	1/155 (1%)
Dopamine-DARPP32 Feedback in cAMP Signaling	3.2E-01	6.21E-03	1/161 (1%)
Tight Junction Signaling	3.08E-01	5.99E-03	1/167 (1%)
Role of NFAT in Regulation of the Immune Response	3.05E-01	5.92E-03	1/169 (1%)
Wnt/β-catenin Signaling	3.05E-01	5.92E-03	1/169 (1%)
Endothelin-1 Signaling	3.01E-01	5.85E-03	1/171 (1%)
CREB Signaling in Neurons	3.01E-01	5.85E-03	1/171 (1%)
RhoGDI Signaling	3E-01	5.81E-03	1/172 (1%)
PPARα/RXRα Activation	2.98E-01	5.78E-03	1/173 (1%)
Ephrin Receptor Signaling	2.98E-01	5.78E-03	1/173 (1%)
Granulocyte Adhesion and Diapedesis	2.91E-01	5.65E-03	1/177 (1%)
Role of NFAT in Cardiac Hypertrophy	2.89E-01	5.62E-03	1/178 (1%)
NRF2-mediated Oxidative Stress Response	2.88E-01	5.59E-03	1/179 (1%)
Production of Nitric Oxide and Reactive Oxygen Species in Macrophages	2.86E-01	5.56E-03	1/180 (1%)
EIF2 Signaling	2.84E-01	5.52E-03	1/181 (1%)
ILK Signaling	2.78E-01	5.41E-03	1/185 (1%)
mTOR Signaling	2.76E-01	5.38E-03	1/186 (1%)
ERK/MAPK Signaling	2.76E-01	5.38E-03	1/186 (1%)
Agranulocyte Adhesion and Diapedesis	2.72E-01	5.29E-03	1/189 (1%)
RAR Activation	2.7E-01	5.26E-03	1/190 (1%)
Superpathway of Inositol Phosphate Compounds	2.7E-01	5.26E-03	1/190 (1%)
Breast Cancer Regulation by Stathmin1	2.69E-01	5.24E-03	1/191 (1%)
Systemic Lupus Erythematosus Signaling	2.37E-01	4.67E-03	1/214 (0%)

Huntington's Disease Signaling	2.19E-01	4.39E-03	1/228 (0%)
Signaling by Rho Family GTPases	2.14E-01	4.29E-03	1/233 (0%)

Table S6. Signaling pathways regulated by the microRNA 1896. Ingenuity Canonical Pathways regulated by mmu-miRNA1896.

Ingenuity Canonical Pathways	-log(p-value)	Ratio	Overlaps with dataset
TWEAK Signaling	4.17E00	8.82E-02	3/34 (9%)
Lymphotoxin β Receptor Signaling	3.57E00	5.56E-02	3/54 (6%)
Induction of Apoptosis by HIV1	3.43E00	5E-02	3/60 (5%)
Small Cell Lung Cancer Signaling	3.22E00	4.23E-02	3/71 (4%)
Death Receptor Signaling	2.89E00	3.26E-02	3/92 (3%)
TNFR2 Signaling	2.68E00	6.9E-02	2/29 (7%)
4-1BB Signaling in T Lymphocytes	2.63E00	6.45E-02	2/31 (6%)
April Mediated Signaling	2.45E00	5.26E-02	2/38 (5%)
Role of PKR in Interferon Induction and Antiviral Response	2.41E00	5E-02	2/40 (5%)
B Cell Activating Factor Signaling	2.41E00	5E-02	2/40 (5%)
TNFR1 Signaling	2.25E00	4.17E-02	2/48 (4%)
CD27 Signaling in Lymphocytes	2.18E00	3.85E-02	2/52 (4%)
Role of PI3K/AKT Signaling in the Pathogenesis of Influenza	2.02E00	3.17E-02	2/63 (3%)
CD40 Signaling	2E00	3.08E-02	2/65 (3%)
Angiopoietin Signaling	2E00	3.08E-02	2/65 (3%)
PXR/RXR Activation	1.99E00	3.03E-02	2/66 (3%)
Serine Biosynthesis	1.94E00	2E-01	1/5 (20%)
Toll-like Receptor Signaling	1.89E00	2.7E-02	2/74 (3%)
Prostate Cancer Signaling	1.81E00	2.44E-02	2/82 (2%)
Superpathway of Serine and Glycine Biosynthesis I	1.79E00	1.43E-01	1/7 (14%)
Apoptosis Signaling	1.74E00	2.25E-02	2/89 (2%)
Antioxidant Action of Vitamin C	1.67E00	2.06E-02	2/97 (2%)
Pancreatic Adenocarcinoma Signaling	1.6E00	1.89E-02	2/106 (2%)
Type I Diabetes Mellitus Signaling	1.58E00	1.85E-02	2/108 (2%)
Androgen Signaling	1.56E00	1.8E-02	2/111 (2%)
Type II Diabetes Mellitus Signaling	1.52E00	1.72E-02	2/116 (2%)
IL-6 Signaling	1.52E00	1.72E-02	2/116 (2%)
PTEN Signaling	1.51E00	1.69E-02	2/118 (2%)
Vitamin-C Transport	1.49E00	7.14E-02	1/14 (7%)
Hereditary Breast Cancer Signaling	1.45E00	1.56E-02	2/128 (2%)
Granzyme B Signaling	1.44E00	6.25E-02	1/16 (6%)
Mismatch Repair in Eukaryotes	1.44E00	6.25E-02	1/16 (6%)

Parkinson's Signaling	1.44E00	6.25E-02	1/16 (6%)
Aryl Hydrocarbon Receptor Signaling	1.38E00	1.44E-02	2/139 (1%)
Endoplasmic Reticulum Stress Pathway	1.32E00	4.76E-02	1/21 (5%)
Tumoricidal Function of Hepatic Natural Killer Cells	1.26E00	4.17E-02	1/24 (4%)
Role of JAK1, JAK2 and TYK2 in Interferon Signaling	1.26E00	4.17E-02	1/24 (4%)
IL-17A Signaling in Gastric Cells	1.25E00	4E-02	1/25 (4%)
IL-15 Production	1.21E00	3.7E-02	1/27 (4%)
Cytotoxic T Lymphocyte-mediated Apoptosis of Target Cells	1.14E00	3.12E-02	1/32 (3%)
MIF-mediated Glucocorticoid Regulation	1.13E00	3.03E-02	1/33 (3%)
IL-9 Signaling	1.12E00	2.94E-02	1/34 (3%)
IL-17A Signaling in Fibroblasts	1.11E00	2.86E-02	1/35 (3%)
Nucleotide Excision Repair Pathway	1.11E00	2.86E-02	1/35 (3%)
Interferon Signaling	1.09E00	2.78E-02	1/36 (3%)
Docosahexaenoic Acid (DHA) Signaling	1.06E00	2.56E-02	1/39 (3%)
MIF Regulation of Innate Immunity	1.04E00	2.44E-02	1/41 (2%)
Role of Osteoblasts, Osteoclasts and Chondrocytes in Rheumatoid Arthritis	1.04E00	9.17E-03	2/218 (1%)
Role of RIG1-like Receptors in Antiviral Innate Immunity	1.02E00	2.33E-02	1/43 (2%)
Role of IL-17F in Allergic Inflammatory Airway Diseases	1.01E00	2.27E-02	1/44 (2%)
iNOS Signaling	1.01E00	2.27E-02	1/44 (2%)
Huntington's Disease Signaling	1.01E00	8.77E-03	2/228 (1%)
Colorectal Cancer Metastasis Signaling	9.81E-01	8.47E-03	2/236 (1%)
Assembly of RNA Polymerase II Complex	9.58E-01	2E-02	1/50 (2%)
Semaphorin Signaling in Neurons	9.42E-01	1.92E-02	1/52 (2%)
Endometrial Cancer Signaling	9.42E-01	1.92E-02	1/52 (2%)
Nur77 Signaling in T Lymphocytes	9.27E-01	1.85E-02	1/54 (2%)
Role of IL-17A in Arthritis	9.27E-01	1.85E-02	1/54 (2%)
Role of CHK Proteins in Cell Cycle Checkpoint Control	9.19E-01	1.82E-02	1/55 (2%)
Wnt/Ca+ pathway	9.12E-01	1.79E-02	1/56 (2%)
Myc Mediated Apoptosis Signaling	8.98E-01	1.72E-02	1/58 (2%)
Retinoic acid Mediated Apoptosis Signaling	8.77E-01	1.64E-02	1/61 (2%)
Glucocorticoid Receptor Signaling	8.72E-01	7.27E-03	2/275 (1%)
Activation of IRF by Cytosolic Pattern Recognition Receptors	8.71E-01	1.61E-02	1/62 (2%)
IL-15 Signaling	8.64E-01	1.59E-02	1/63 (2%)
Estrogen-Dependent Breast Cancer Signaling	8.64E-01	1.59E-02	1/63 (2%)
IL-17A Signaling in Airway Cells	8.58E-01	1.56E-02	1/64 (2%)
Non-Small Cell Lung Cancer Signaling	8.52E-01	1.54E-02	1/65 (2%)
Hypoxia Signaling in the Cardiovascular System	8.52E-01	1.54E-02	1/65 (2%)
Erythropoietin Signaling	8.39E-01	1.49E-02	1/67 (1%)

IL-10 Signaling	8.33E-01	1.47E-02	1/68 (1%)
Growth Hormone Signaling	8.28E-01	1.45E-02	1/69 (1%)
Role of Macrophages, Fibroblasts and Endothelial Cells in Rheumatoid Arthritis	8.23E-01	6.78E-03	2/295 (1%)
Melatonin Signaling	8.22E-01	1.43E-02	1/70 (1%)
PEDF Signaling	8.16E-01	1.41E-02	1/71 (1%)
IL-17 Signaling	8.1E-01	1.39E-02	1/72 (1%)
JAK/Stat Signaling	8.1E-01	1.39E-02	1/72 (1%)
LPS-stimulated MAPK Signaling	8.05E-01	1.37E-02	1/73 (1%)
NF-κB Activation by Viruses	8.05E-01	1.37E-02	1/73 (1%)
TREM1 Signaling	7.94E-01	1.33E-02	1/75 (1%)
BMP signaling pathway	7.94E-01	1.33E-02	1/75 (1%)
HER-2 Signaling in Breast Cancer	7.89E-01	1.32E-02	1/76 (1%)
OX40 Signaling Pathway	7.89E-01	1.32E-02	1/76 (1%)
Role of BRCA1 in DNA Damage Response	7.79E-01	1.28E-02	1/78 (1%)
Acute Myeloid Leukemia Signaling	7.74E-01	1.27E-02	1/79 (1%)
Regulation of IL-2 Expression in Activated and Anergic T Lymphocytes	7.74E-01	1.27E-02	1/79 (1%)
Ceramide Signaling	7.69E-01	1.25E-02	1/80 (1%)
Altered T Cell and B Cell Signaling in Rheumatoid Arthritis	7.4E-01	1.16E-02	1/86 (1%)
RANK Signaling in Osteoclasts	7.31E-01	1.14E-02	1/88 (1%)
UVA-Induced MAPK Signaling	7.31E-01	1.14E-02	1/88 (1%)
Crosstalk between Dendritic Cells and Natural Killer Cells	7.27E-01	1.12E-02	1/89 (1%)
IL-1 Signaling	7.18E-01	1.1E-02	1/91 (1%)
Chronic Myeloid Leukemia Signaling	7.14E-01	1.09E-02	1/92 (1%)
PPAR Signaling	7.09E-01	1.08E-02	1/93 (1%)
IGF-1 Signaling	6.93E-01	1.03E-02	1/97 (1%)
T Cell Receptor Signaling	6.93E-01	1.03E-02	1/97 (1%)
Amyotrophic Lateral Sclerosis Signaling	6.89E-01	1.02E-02	1/98 (1%)
Molecular Mechanisms of Cancer	6.84E-01	5.51E-03	2/363 (1%)
HIF1α Signaling	6.77E-01	9.9E-03	1/101 (1%)
Rac Signaling	6.7E-01	9.71E-03	1/103 (1%)
iCOS-iCOSL Signaling in T Helper Cells	6.59E-01	9.43E-03	1/106 (1%)
NGF Signaling	6.55E-01	9.35E-03	1/107 (1%)
fMLP Signaling in Neutrophils	6.52E-01	9.26E-03	1/108 (1%)
Renin-Angiotensin Signaling	6.52E-01	9.26E-03	1/108 (1%)
Sphingosine-1-phosphate Signaling	6.48E-01	9.17E-03	1/109 (1%)
CD28 Signaling in T Helper Cells	6.24E-01	8.62E-03	1/116 (1%)
PKCθ Signaling in T Lymphocytes	6.24E-01	8.62E-03	1/116 (1%)
Gα12/13 Signaling	6.21E-01	8.55E-03	1/117 (1%)
p38 MAPK Signaling	6.21E-01	8.55E-03	1/117 (1%)
HMGB1 Signaling	6.15E-01	8.4E-03	1/119 (1%)
P2Y Purigenic Receptor Signaling Pathway	6.15E-01	8.4E-03	1/119 (1%)

LXR/RXR Activation	6.08E-01	8.26E-03	1/121 (1%)
Atherosclerosis Signaling	6.05E-01	8.2E-03	1/122 (1%)
PI3K/AKT Signaling	6.02E-01	8.13E-03	1/123 (1%)
Role of Pattern Recognition Receptors in Recognition of Bacteria and Viruses	5.99E-01	8.06E-03	1/124 (1%)
PI3K Signaling in B Lymphocytes	5.9E-01	7.87E-03	1/127 (1%)
GNRH Signaling	5.87E-01	7.81E-03	1/128 (1%)
Estrogen Receptor Signaling	5.87E-01	7.81E-03	1/128 (1%)
Insulin Receptor Signaling	5.76E-01	7.58E-03	1/132 (1%)
IL-12 Signaling and Production in Macrophages	5.73E-01	7.52E-03	1/133 (1%)
Adipogenesis pathway	5.73E-01	7.52E-03	1/133 (1%)
Relaxin Signaling	5.68E-01	7.41E-03	1/135 (1%)
eNOS Signaling	5.51E-01	7.09E-03	1/141 (1%)
Regulation of eIF4 and p70S6K Signaling	5.41E-01	6.9E-03	1/145 (1%)
Gαq Signaling	5.36E-01	6.8E-03	1/147 (1%)
Aldosterone Signaling in Epithelial Cells	5.24E-01	6.58E-03	1/152 (1%)
Tec Kinase Signaling	5.15E-01	6.41E-03	1/156 (1%)
Hepatic Cholestasis	5.06E-01	6.25E-03	1/160 (1%)
Tight Junction Signaling	4.9E-01	5.99E-03	1/167 (1%)
Acute Phase Response Signaling	4.86E-01	5.92E-03	1/169 (1%)
Mitochondrial Dysfunction	4.86E-01	5.92E-03	1/169 (1%)
Role of NFAT in Regulation of the Immune Response	4.86E-01	5.92E-03	1/169 (1%)
Endothelin-1 Signaling	4.82E-01	5.85E-03	1/171 (1%)
CREB Signaling in Neurons	4.82E-01	5.85E-03	1/171 (1%)
NF-κB Signaling	4.82E-01	5.85E-03	1/171 (1%)
PPARα/RXRα Activation	4.78E-01	5.78E-03	1/173 (1%)
Dendritic Cell Maturation	4.76E-01	5.75E-03	1/174 (1%)
B Cell Receptor Signaling	4.76E-01	5.75E-03	1/174 (1%)
AMPK Signaling	4.68E-01	5.62E-03	1/178 (1%)
Production of Nitric Oxide and Reactive Oxygen Species in Macrophages	4.64E-01	5.56E-03	1/180 (1%)
EIF2 Signaling	4.62E-01	5.52E-03	1/181 (1%)
Hepatic Fibrosis / Hepatic Stellate Cell Activation	4.6E-01	5.49E-03	1/182 (1%)
IL-8 Signaling	4.58E-01	5.46E-03	1/183 (1%)
Regulation of the Epithelial-Mesenchymal Transition Pathway	4.56E-01	5.43E-03	1/184 (1%)
ILK Signaling	4.54E-01	5.41E-03	1/185 (1%)
mTOR Signaling	4.52E-01	5.38E-03	1/186 (1%)
ERK/MAPK Signaling	4.52E-01	5.38E-03	1/186 (1%)
Thrombin Signaling	4.47E-01	5.29E-03	1/189 (1%)
RAR Activation	4.45E-01	5.26E-03	1/190 (1%)
Systemic Lupus Erythematosus Signaling	4.04E-01	4.67E-03	1/214 (0%)
Signaling by Rho Family GTPases	3.76E-01	4.29E-03	1/233 (0%)
Phospholipase C Signaling	3.73E-01	4.26E-03	1/235 (0%)
Protein Ubiquitination Pathway	3.47E-01	3.92E-03	1/255 (0%)

G-Protein Coupled Receptor Signaling	3.45E-01	3.91E-03	1/256 (0%)
Xenobiotic Metabolism Signaling	3.3E-01	3.72E-03	1/269 (0%)
Protein Kinase A Signaling	2.28E-01	2.63E-03	1/380 (0%)

Table S7. Signaling pathways regulated by the microRNA 874. Ingenuity Canonical Pathways regulated by mmu-miRNA 874.

**Ingenuity Canonical Pathways**	**-log(p-value)**	**Ratio**	**Overlaps with dataset**
L-cysteine Degradation III	1.91E00	5E-01	1/2 (50%)
Epithelial Adherens Junction Signaling	1.89E00	2.74E-02	4/146 (3%)
Thiosulfate Disproportionation III (Rhodanese)	1.74E00	3.33E-01	1/3 (33%)
Sertoli Cell-Sertoli Cell Junction Signaling	1.61E00	2.25E-02	4/178 (2%)
Glycoaminoglycan-protein Linkage Region Biosynthesis	1.37E00	1.43E-01	1/7 (14%)
PEDF Signaling	1.15E00	2.82E-02	2/71 (3%)
Hematopoiesis from Multipotent Stem Cells	1.15E00	8.33E-02	1/12 (8%)
Germ Cell-Sertoli Cell Junction Signaling	1.12E00	1.89E-02	3/159 (2%)
TR/RXR Activation	1.01E00	2.35E-02	2/85 (2%)
Differential Regulation of Cytokine Production in Macrophages and T Helper Cells by IL-17A and IL-17F	9.78E-01	5.56E-02	1/18 (6%)
DNA Methylation and Transcriptional Repression Signaling	9.35E-01	5E-02	1/20 (5%)
Differential Regulation of Cytokine Production in Intestinal Epithelial Cells by IL-17A and IL-17F	8.78E-01	4.35E-02	1/23 (4%)
D-myo-inositol (1,4,5)-Trisphosphate Biosynthesis	8.28E-01	3.85E-02	1/26 (4%)
Cell Cycle Control of Chromosomal Replication	8.13E-01	3.7E-02	1/27 (4%)
Intrinsic Prothrombin Activation Pathway	7.99E-01	3.57E-02	1/28 (4%)
Sonic Hedgehog Signaling	7.71E-01	3.33E-02	1/30 (3%)
Atherosclerosis Signaling	7.61E-01	1.64E-02	2/122 (2%)
TWEAK Signaling	7.22E-01	2.94E-02	1/34 (3%)
Netrin Signaling	6.69E-01	2.56E-02	1/39 (3%)
Thyroid Cancer Signaling	6.59E-01	2.5E-02	1/40 (3%)
iNOS Signaling	6.23E-01	2.27E-02	1/44 (2%)
Serotonin Receptor Signaling	6.23E-01	2.27E-02	1/44 (2%)
Aldosterone Signaling in Epithelial Cells	6.19E-01	1.32E-02	2/152 (1%)
MSP-RON Signaling Pathway	6.06E-01	2.17E-02	1/46 (2%)
Hematopoiesis from Pluripotent Stem Cells	5.98E-01	2.13E-02	1/47 (2%)
Phototransduction Pathway	5.54E-01	1.89E-02	1/53 (2%)

Role of Cytokines in Mediating Communication between Immune Cells	5.47E-01	1.85E-02	1/54 (2%)
Chondroitin Sulfate Biosynthesis	5.47E-01	1.85E-02	1/54 (2%)
RhoGDI Signaling	5.43E-01	1.16E-02	2/172 (1%)
EGF Signaling	5.33E-01	1.79E-02	1/56 (2%)
Heparan Sulfate Biosynthesis	5.27E-01	1.75E-02	1/57 (2%)
Dermatan Sulfate Biosynthesis	5.27E-01	1.75E-02	1/57 (2%)
Granulocyte Adhesion and Diapedesis	5.26E-01	1.13E-02	2/177 (1%)
Hypoxia Signaling in the Cardiovascular System	4.8E-01	1.54E-02	1/65 (2%)
Neurotrophin/TRK Signaling	4.69E-01	1.49E-02	1/67 (1%)
Remodeling of Epithelial Adherens Junctions	4.64E-01	1.47E-02	1/68 (1%)
T Helper Cell Differentiation	4.59E-01	1.45E-02	1/69 (1%)
Growth Hormone Signaling	4.59E-01	1.45E-02	1/69 (1%)
LPS-stimulated MAPK Signaling	4.39E-01	1.37E-02	1/73 (1%)
NF-κB Activation by Viruses	4.39E-01	1.37E-02	1/73 (1%)
IL-4 Signaling	4.39E-01	1.37E-02	1/73 (1%)
PDGF Signaling	4.21E-01	1.3E-02	1/77 (1%)
Actin Cytoskeleton Signaling	4.2E-01	9.35E-03	2/214 (1%)
VDR/RXR Activation	4.17E-01	1.28E-02	1/78 (1%)
Dopamine Receptor Signaling	4.17E-01	1.28E-02	1/78 (1%)
Prostate Cancer Signaling	4E-01	1.22E-02	1/82 (1%)
Cardiac Hypertrophy Signaling	3.98E-01	8.97E-03	2/223 (1%)
HIPPO signaling	3.84E-01	1.16E-02	1/86 (1%)
Regulation of Actin-based Motility by Rho	3.73E-01	1.12E-02	1/89 (1%)
Apoptosis Signaling	3.73E-01	1.12E-02	1/89 (1%)
PPAR Signaling	3.59E-01	1.08E-02	1/93 (1%)
IGF-1 Signaling	3.45E-01	1.03E-02	1/97 (1%)
Cholecystokinin/Gastrin-mediated Signaling	3.33E-01	9.9E-03	1/101 (1%)
Protein Ubiquitination Pathway	3.3E-01	7.84E-03	2/255 (1%)
Rac Signaling	3.26E-01	9.71E-03	1/103 (1%)
IL-6 Signaling	2.9E-01	8.62E-03	1/116 (1%)
14-3-3-mediated Signaling	2.88E-01	8.55E-03	1/117 (1%)
p38 MAPK Signaling	2.88E-01	8.55E-03	1/117 (1%)
Gαi Signaling	2.8E-01	8.33E-03	1/120 (1%)
phagosome maturation	2.8E-01	8.33E-03	1/120 (1%)
RhoA Signaling	2.78E-01	8.26E-03	1/121 (1%)
Gustation Pathway	2.78E-01	8.26E-03	1/121 (1%)
Cellular Effects of Sildenafil (Viagra)	2.62E-01	7.81E-03	1/128 (1%)
Insulin Receptor Signaling	2.53E-01	7.58E-03	1/132 (1%)
Human Embryonic Stem Cell Pluripotency	2.51E-01	7.52E-03	1/133 (1%)
3-phosphoinositide Biosynthesis	2.13E-01	6.54E-03	1/153 (1%)
Gap Junction Signaling	2.09E-01	6.45E-03	1/155 (1%)

Table S8. Signaling pathways regulated by the microRNA 296-5p. Ingenuity Canonical Pathways regulated by mmu-miRNA 296-5p.

	Control diet	Obesity-inducing diet (OID)
Energy from fat (%)	**17.2**	**58.5**
Energy from protein (%)	**18.6**	**18.8**
Energy from carbohydrate (%)	**63.0**	**22.8**
Kcal/gram	**3.8**	**5.4**

Table S9. **Composition and nutrient content in control and obesity-inducing (OID) diets.** Control and OID are AIN93G-based diets containing either 17% or *58* % energy from fat (sources: corn oil+ lard), respectively.

Name	Maker	Cat. Number	Dilution
Actin	Santa Cruz	7210	1:1000
Tubulin	Millipore	AB3201	1:2000
DNMT3A	Cell Signaling	2160	1:1000
VEGF-A	Santa Cruz	sc-152	1:1000 or 1:25 (IHC)
ERα	Santa Cruz	sc-7207	1:1000
MAPK	Cell Signaling	9102	1:1000
Phopo-MAPK	Cell Signaling	9101	1:1000
HIF-1α	Novus Biologicals	100-134	1:500 or 1:25 (IHC)
CD31	Abcam	Ab-28364	1:25 (IHC)

Table S10. Specification for antibodies used in western-blot and immunohistochemistry (IHC) experiments.
